# Facile Synthesis for Benzo-1,4-Oxazepine Derivatives by Tandem Transformation of C-N Coupling/C-H Carbonylation

**DOI:** 10.3390/molecules22010053

**Published:** 2016-12-30

**Authors:** Xiaojia Zhao, Jiong Zhang, Zeqin Zheng, Runsheng Xu

**Affiliations:** Department of Biology and Environment, Zhejiang A&F University, Shaoxing 311800, Zhejiang, China; 18267573167m@sina.cn (X.Z.); a63214955@sohu.com (J.Z.); 13282210629@163.com (Z.Z.)

**Keywords:** benzo-1,4-oxazepine, copper catalyst, tandem transformation, C-N coupling, C-H carbonylation

## Abstract

A tandem transformation of C-N coupling/C-H carbonylation has been developed for the synthesis of benzo-1,4-oxazepine pharmaceutically derivatives. Notably, this reaction was accomplished by various phenylamine with ally halides under carbon dioxide atmosphere employing 2-(2-dimethylamino-vinyl)-1*H*-inden-1-olcatalyzed. Furthermore, under the optimized conditions, various benzo-1,4-oxazepine derivatives were obtained in good yields. Finally, a plausible Cu^I^/Cu^III^ mechanism of C-N coupling/C-H carbonylation transformation was proposed.

## 1. Introduction

**Scheme 1:**
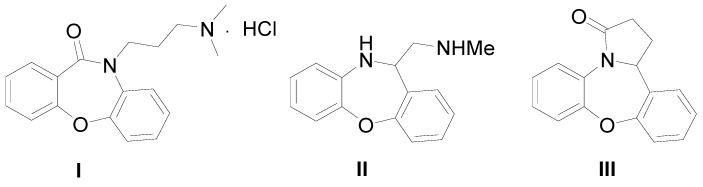
The important benzo-1,4-oxazepine derivatives.

The heterocycle benzoxazepines are privileged scaffolds in natural biologically products [1,2,3,4], pharmaceutical chemistry [5,6] and functionalized materials [7,8,9,10]. As such, Sintamilv (**I**) is an efficient antidepressant [11]; H1 receptor antagonist (**II**) is a selective antihistaminic agent [12]; and Sintamil (**III**) is a benzoxazepine analogue (Scheme 1) [13]. Furthermore, the therapeutic applications of benzoxazepines are for the central nervous system, along with anti-breast cancer activity and inhibitors of HIV [14,15].

Currently, the challenge in organic synthesis is developing an efficient and eco-friendly protocol, especially in the area of drug discovery and natural products. Benzoxazepines are generally synthesized by condensation of 2-aryloxyethylamines with 2-formylbenzoic acid [16]. Others have also been synthesized from amides [17] and amino acids [18,19]. However, most of these methodologies are associated with several drawbacks, such as low synthetic efficiency and sensitivity. Thus, a remarkable gap remains in the search of economical synthesis methods. Tandem transformation is one of the most effective ways to achieve this goal. Considering the above points, herein we report the tandem reaction green protocol for the synthesis of benzo-1,4-oxazepine pharmaceutical derivatives.

The reaction conditions were screened based on a model reaction of phenylamine **1a** and (1-chloro-vinyl)-benzene **2a** (Table 1). The ligands were mainly based on the derivatives of 2-(2-dimethylamino-vinyl)-1*H*-inden-1-ol. It was discovered that ligand **L1** was the ideal choice for this transformation (Entries 5–10). CuI exhibited superior catalytic efficiency over all other examined Cu^I^ catalysts (Entries 1–5), and Cs_2_CO_3_ turned out to be the proper base additive (Entries 11–12). Meanwhile, the reaction temperature was 100 °C (Entries 15–16).

With the optimal conditions established, the reaction scope was further investigated. A wide array of phenylamine **1** and ally halide **2** was subjected to this reaction in moderate to good yields (Table 2). Phenylamine derivatives bearing either an electron-withdrawing or electron-donating group reacted smoothly with **2**. This transformation is applicable for *para*-substituted phenylamines. Chloroethylene bearing an electron-donating group showed better reactivity than those with an electron-withdrawing group (All the product spectrums, please see Supplementary Materials).

Interestingly, we found that 1-bromo-cyclohexene **4** has also been rapidly synthesized in good yields, and the results are summarized in Table 3. In addition, the reaction works well for both bearing electron-donating and electron-withdrawing groups.

**Scheme 2:**
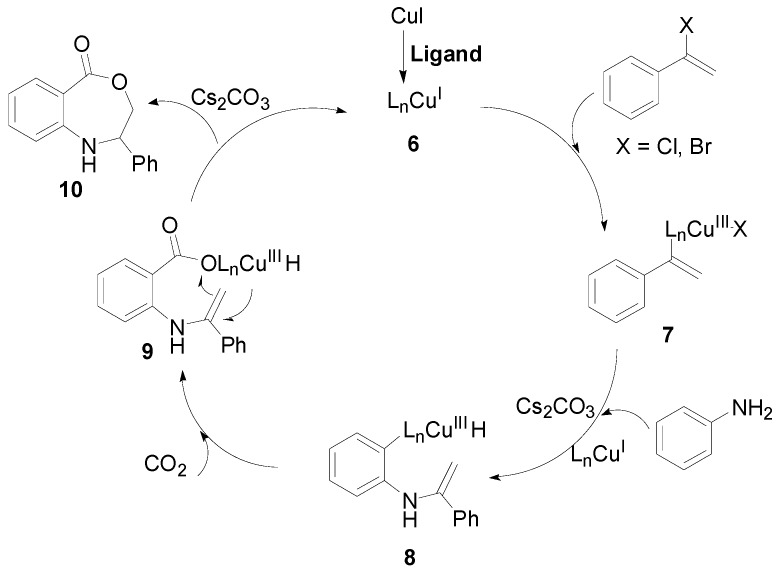
A plausible mechanism of the catalytic cycle.

On the basis of the above experimental results, we tentatively proposed a reaction mechanism as shown in Scheme 2. At the beginning, Cu^I^ activate **6** was been formed through copper iodide coordinating with ligand. Next, complex **6** reacted with vinyl halides by oxidative addition produced a Cu^III^ complex **7**. The complex **7** reacted with aniline obtained the key intermediate complex **8** [20,21]. Selective *ortho*-carbonylation of the phenylamine was determined by Complex **9**. Through the reductive elimination of Complex **9**, Complex **10** was obtained, which regenerates Complex **6** for the next catalytic cycle [22,23]. However, how the ligand promotes this transformation is a part of ongoing study.

## 2. Results and Discussion


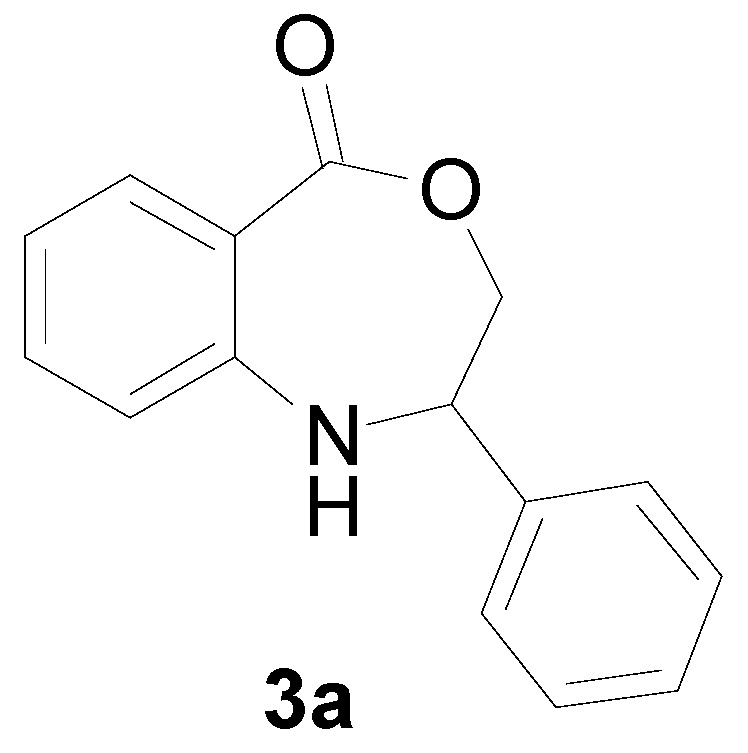


*2-Phenyl-2,3-dihydro-1H-benzo[e][1,4]oxazepin-5-one* (**3a**): A mixture of phenylamine **1a** (0.5 mmol, 46.5 mg), (1-chloro-vinyl)-benzene **2a** (0.6 mmol, 83.4 mg), CuI (10 mol %, 9.5 mg), **L1** (10 mol %, 20.1 mg) and Cs_2_CO_3_ (2 equiv., 325.8 mg) in DMSO (4 mL) was stirred in CO_2_ at 100 °C for 10 h. After completion of the reaction, the mixture was quenched with saturated salt water (10 mL); the solution was extracted with ethyl acetate (3 × 10 mL). The organic layers were combined and dried over sodium sulfate. The pure product was obtained by flash column chromatography on silica gel to afford **3a** 96.8 mg in 81% yield. The spectroscopic data of all of the products are presented below. Yellowish oil. ^1^H-NMR (400 MHz, CDCl_3_): 7.63 (m, 1H), 7.43 (br, 1H), 7.08–7.43 (m, 8H), 5.07 (dd, *J* = 8.0, 5.7 Hz, 1H), 4.08 (dd, *J* = 12.3, 8.0 Hz, 1H), 3.96 (dd, *J* = 12.3, 5.6 Hz, 1H); ^13^C-NMR (100 MHz, CDCl_3_): 168.3, 147.7, 139.1, 132.9, 130.3, 128.6, 127.5, 126.6, 117.8, 116.4, 109.1, 77.6, 60.2; EIMS (*m*/*z*): 239 [M^+^]; Anal. Calcd. for C_15_H_13_NO_2_: C, 75.30; H, 5.48; N, 5.85; Found: C, 75.62; H, 5.13; N, 5.68.


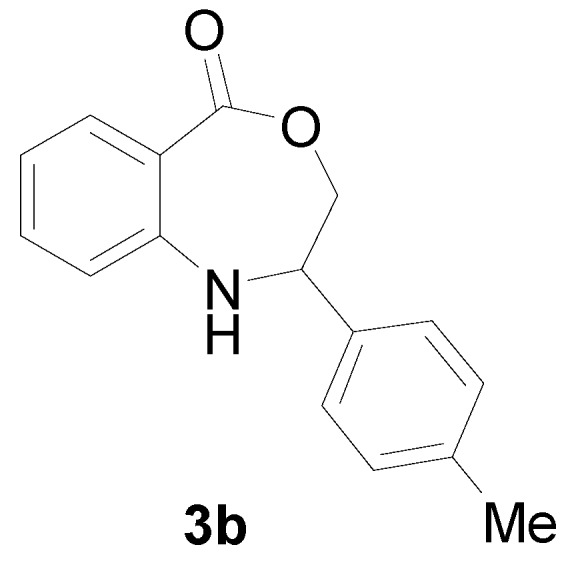


*2-p-Tolyl-2,3-dihydro-1H-benzo[e][1,4] xazepine-5-one* (**3b**): Yellowish oil. ^1^H-NMR (400 MHz, CDCl_3_): 7.61 (m, 1H), 7.44 (br, 1H), 7.04–7.31 (m, 7H), 5.07 (dd, *J* = 8.0, 5.7 Hz, 1H), 4.07 (dd, *J* = 12.3, 8.0 Hz, 1H), 3.95 (dd, *J* = 12.3, 5.7 Hz, 1H), 2.39 (s, 3H); ^13^C-NMR (100 MHz, CDCl_3_): 168.6, 147.8, 138.3, 135.3, 132.3, 130.5, 128.1, 127.6, 118.2, 115.9, 109.5, 77.5, 60.3, 25.2; EIMS (*m*/*z*): 253 [M^+^]; Anal. Calcd. for C_16_H_15_NO_2_: C, 75.87; H, 5.97; N, 5.53; Found: C, 75.50; H, 6.20; N, 5.88.


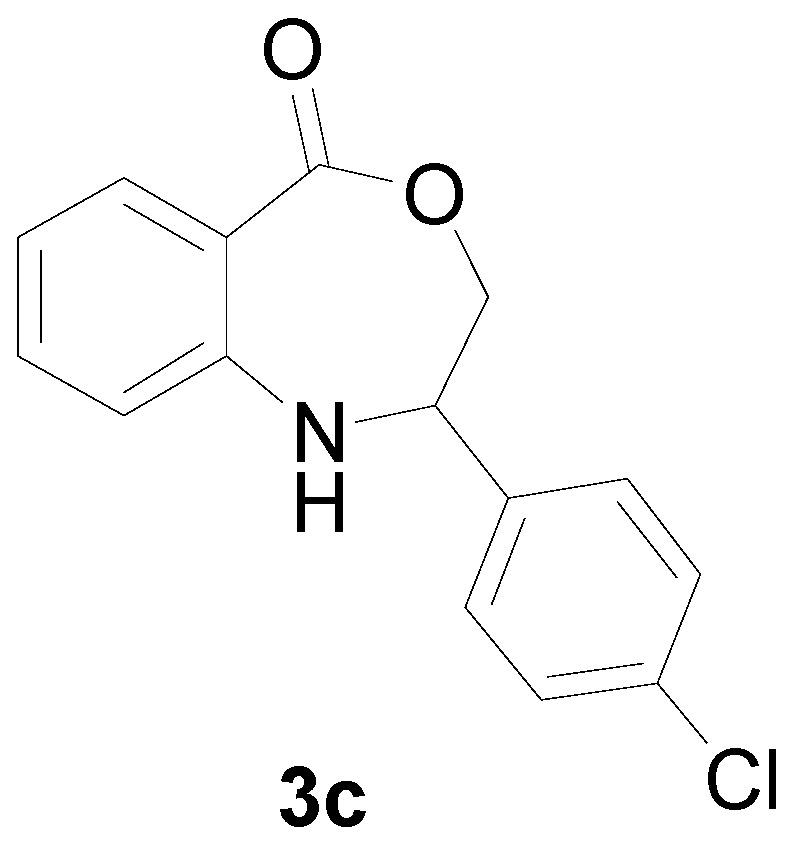


*2-(4-Chloro-phenyl)-2,3-dihydro-1H-benzo[e][1,4] xazepine-5-one* (**3c**): Yellowish oil. ^1^H-NMR (400 MHz, CDCl_3_): 7.64 (m, 1H), 7.47 (br, 1H), 7.07–7.48 (m, 7H), 5.08 (dd, *J* = 8.1, 5.6 Hz, 1H), 4.09 (dd, *J* = 12.3, 8.1 Hz, 1H), 3.95 (dd, *J* = 12.3, 5.6 Hz, 1H); ^13^C-NMR (100 MHz, CDCl_3_): 168.3, 147.7, 139.3, 133.3, 132.4, 130.5, 128.6, 127.8, 118.4, 116.3, 110.1, 77.3, 60.9;EIMS (*m*/*z*): 273 [M^+^]; Anal. Calcd. for C_15_H_12_ClNO_2_: C, 65.82; H, 4.42; N, 5.12; Found: C, 65.51; H, 4.61; N, 5.33.


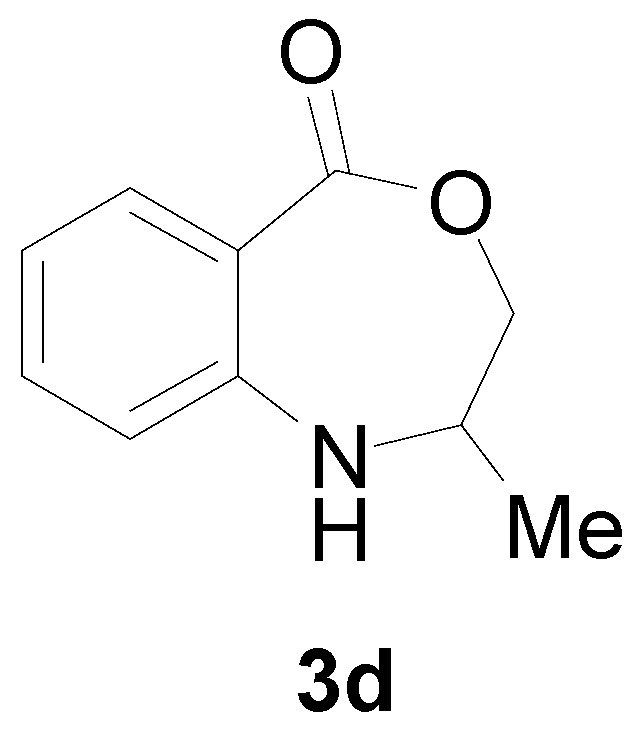


*2-Methyl-2,3-dihydro-1H-benzo[e][1,4]oxazepin-5-one* (**3d**): Yellowish oil. ^1^H-NMR (400 MHz, CDCl_3_): 7.62 (m, 1H), 7.42 (br, 1H), 7.05–7.21 (m, 3H), 4.58 (dd, *J* = 12.3, 8.0 Hz, 1H), 3.96 (dd, *J* = 12.2, 5.6 Hz, 1H), 3.12–3.71 (m, 1H), 1.35 (d, *J* = 7.1 Hz, 3H); ^13^C-NMR (100 MHz, CDCl_3_): 168.2, 147.3, 132.8, 130.4, 118.7, 116.6, 109.7, 77.1, 53.1, 18.2; EIMS (*m*/*z*): 177.08 [M^+^]; Anal. Calcd. for C_10_H_11_NO_2_: C, 67.78; H, 6.26; N, 7.90; Found: C, 68.14; H, 6.55; N, 7.53.


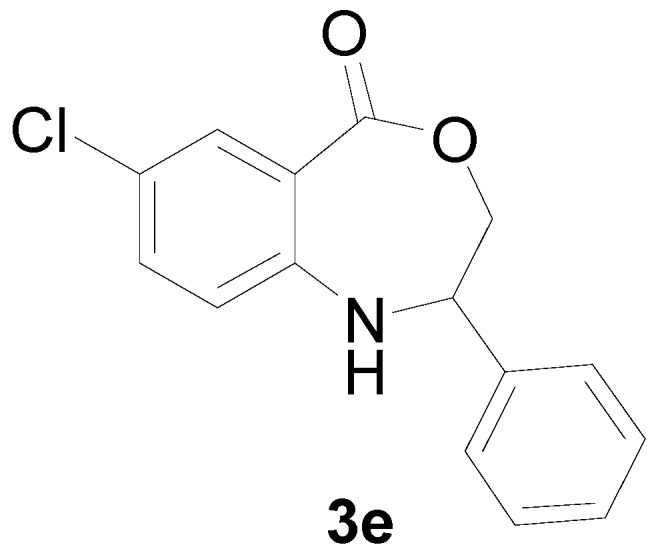


*7-Chloro-2-phenyl-2,3-dihydro-1H-benzo[e][1,4]oxazepin-5-one* (**3e**): Yellowish oil. ^1^H-NMR (400 MHz, CDCl_3_): 7.63 (m, 1H), 7.43 (br, 1H), 7.10–7.46 (m, 7H), 5.08 (dd, *J* = 8.1, 5.6 Hz, 1H), 4.10 (dd, *J* = 12.4, 8.1 Hz, 1H), 3.97 (dd, *J* = 12.4, 5.6 Hz, 1H); ^13^C-NMR (100 MHz, CDCl_3_): 168.3, 147.4, 139.5, 133.2, 130.2, 128.7, 127.5, 126.8, 123.8, 115.4, 109.2, 77.5, 60.2; EIMS (*m*/*z*): 273 [M^+^]; Anal. Calcd. for C_15_H_12_ClNO_2_: C, 65.82; H, 4.42; N, 5.12; Found: C, 65.70; H, 4.61; N, 5.44.


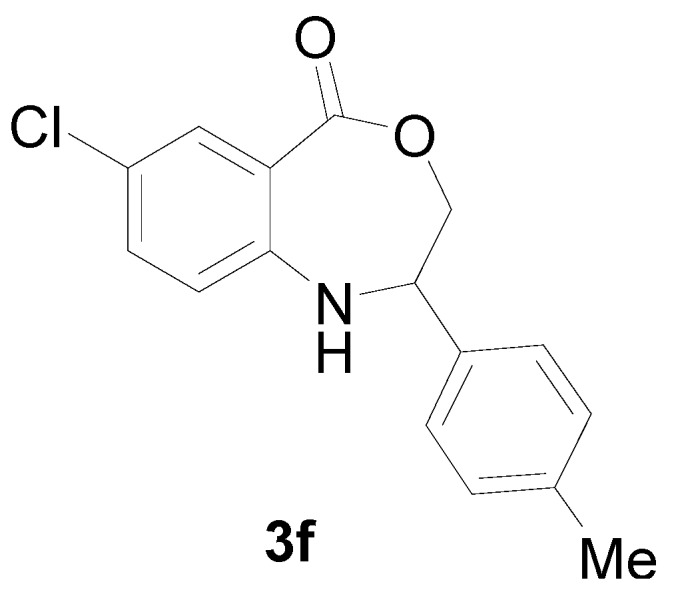


*7-Chloro-2-p-tolyl-2,3-dihydro-1H-benzo[e][1,4]oxazepin-5-one* (**3f**): Yellowish oil. ^1^H-NMR (400 MHz, CDCl_3_): 7.64 (m, 1H), 7.43 (br, 1H), 7.07–7.38 (m, 6H), 5.08 (dd, *J* = 8.1, 5.9 Hz, 1H), 4.10 (dd, *J* = 12.4, 8.1 Hz, 1H), 3.96 (dd, *J* = 12.4, 5.9 Hz, 1H), 2.40 (s, 3H); ^13^C-NMR (100 MHz, CDCl_3_): 168.2, 147.1, 139.2, 135.8, 133.4, 130.5, 128.7, 126.9, 123.5, 115.5, 109.3, 77.2, 60.4, 25.7; EIMS (*m*/*z*): 287.07 [M^+^]; Anal. Calcd. for C_16_H_14_ClNO_2_: C, 66.79; H, 4.90; N, 4.87; Found: C, 66.95; H, 4.63; N, 5.23.


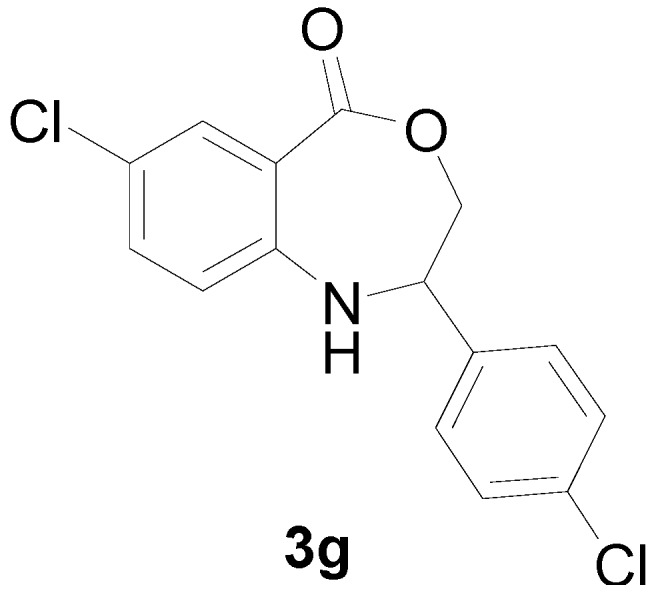


*7-Chloro-2-(4-chloro-phenyl)-2,3-dihydro-1H-benzo[e][1,4]oxazepin-5-one* (**3g**): Yellowish oil. ^1^H-NMR (400 MHz, CDCl_3_): 7.66 (m, 1H), 7.46 (br, 1H), 7.09–7.50 (m, 6H), 5.10 (dd, *J* = 8.2, 5.6 Hz, 1H), 4.11 (dd, *J* = 12.4, 8.2 Hz, 1H), 3.96 (dd, *J* = 12.4, 5.6 Hz, 1H); ^13^C-NMR (100 MHz, CDCl_3_): 168.2, 147.4, 139.6, 133.2, 131.8, 130.2, 128.9, 126.7, 123.8, 115.2, 109.6, 77.5, 60.3; EIMS (*m*/*z*): 307 [M^+^]; Anal. Calcd. for C_15_H_11_Cl_2_NO_2_: C, 58.46; H, 3.60; N, 4.55; Found: C, 58.23; H, 3.92; N, 4.67.


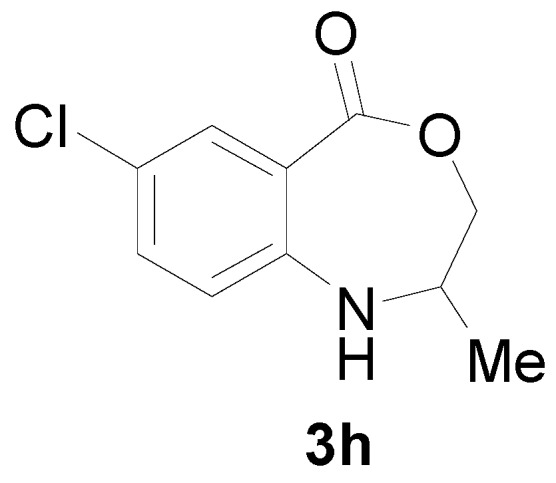


*7-Chloro-2-methyl-2,3-dihydro-1H-benzo[e][1,4]oxazepin-5-one* (**3h**): Yellowish oil. ^1^H-NMR (400 MHz, CDCl_3_): 7.64 (m, 1H), 7.45 (br, 1H), 7.06–7.23 (m, 2H), 4.6 (dd, *J* = 12.2, 8.1 Hz, 1H), 3.98 (dd, *J* = 12.2, 5.6 Hz, 1H), 3.12–3.71 (m, 1H), 1.36 (d, *J* = 7.2 Hz, 3H); ^13^C-NMR (100 MHz, CDCl_3_): 168.5, 147.3, 133.1, 130.2, 123.1, 116.8, 109.3, 77.5, 53.4, 18.3; EIMS (*m*/*z*): 211 [M^+^]; Anal. Calcd. for C_10_H_10_ClNO_2_: C, 56.75; H, 4.76; N, 6.62; Found: C, 56.89; H, 5.18; N, 6.34.


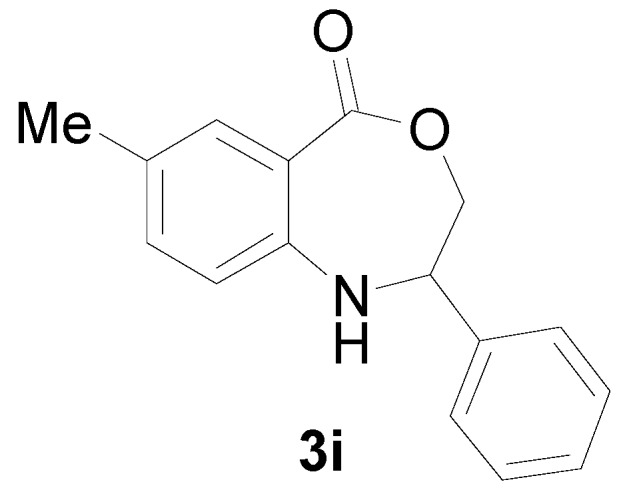


*7-Methyl-2-phenyl-2,3-dihydro-1H-benzo[e][1,4]oxazepin-5-one* (**3i**): Yellowish oil. ^1^H-NMR (400 MHz, CDCl_3_): 7.58 (m, 1H), 7.41 (br, 1H), 7.06–7.40 (m, 7H), 5.00 (dd, *J* = 8.0, 5.6 Hz, 1H), 4.06 (dd, *J* = 12.2, 8.0 Hz, 1H), 3.92 (dd, *J* = 12.2, 5.6 Hz, 1H), 2.40 (s, 3H). ^13^C-NMR (100 MHz, CDCl_3_): 168.5, 147.2, 139.4, 133.3, 130.8, 128.9, 127.7, 126.9, 126.2, 116.7, 109.3, 77.8, 60.3, 25.3; EIMS (*m*/*z*): 253 [M^+^]; Anal. Calcd. for C_16_H_15_NO_2_: C, 75.87; H, 5.97; N, 5.53; Found: C, 75.65; H, 6.28; N, 5.33.


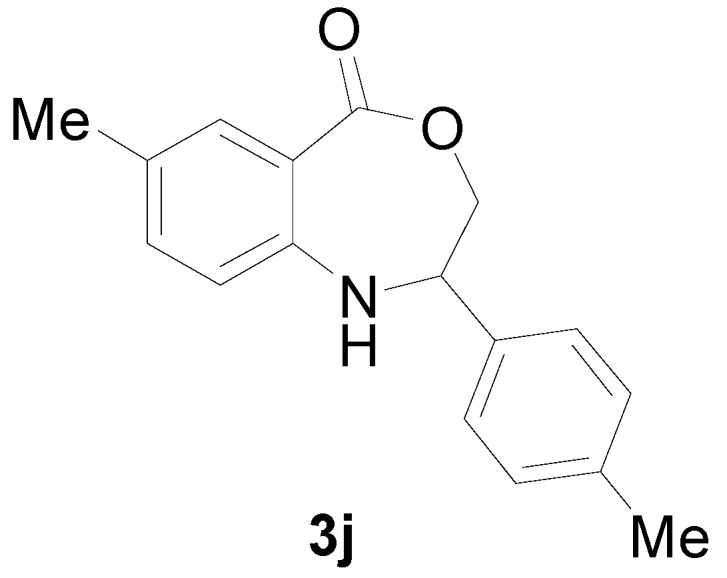


*7-Methyl-2-p-tolyl-2,3-dihydro-1H-benzo[e][1,4]oxazepin-5-one* (**3j**): Yellowish oil. ^1^H-NMR (400 MHz, CDCl_3_): 7.56 (m, 1H), 7.44 (br, 1H), 7.06–7.36 (m, 6H), 4.98 (dd, *J* = 7.9, 5.6 Hz, 1H), 4.02 (dd, *J* = 12.2, 7.9 Hz, 1H), 3.90 (dd, *J* = 12.2, 5.6 Hz, 1H), 2.39 (s, 6H); ^13^C-NMR (100 MHz, CDCl_3_): 168.2, 147.5, 138.3, 135.1, 132.4, 130.8, 128.8, 127.5, 126.2, 116.2, 109.1, 77.2, 60.5, 25.8, 25.3; EIMS (*m*/*z*): 267 [M^+^]; Anal. Calcd. for C_17_H_17_NO_2_: C, 76.38; H, 6.41; N, 5.24; Found: C, 76.69; H, 6.24; N, 5.53.


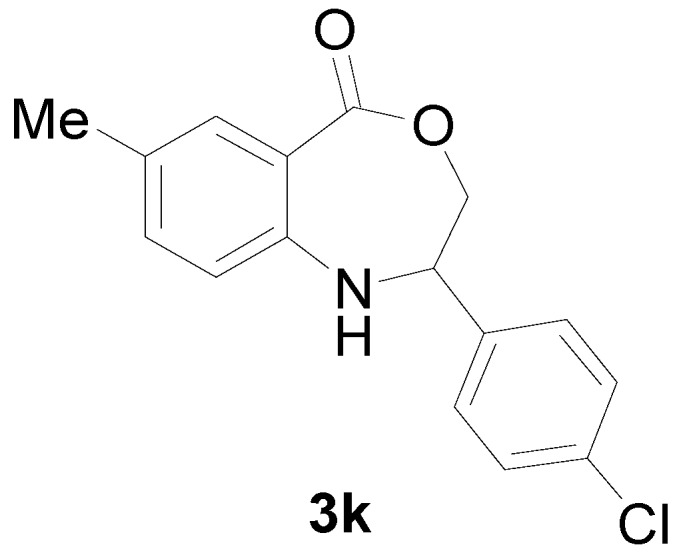


*2-(4-Chloro-phenyl)-7-methyl-2,3-dihydro-1H-benzo[e][1,4]oxazepin-5-one* (**3k**): Yellowish oil. ^1^H-NMR (400 MHz, CDCl_3_): 7.60 (m, 1H), 7.47 (br, 1H), 7.06-7.44 (m, 6H), 5.08 (dd, *J* = 8.0, 5.7 Hz, 1H), 4.10 (dd, *J* = 12.2, 8.0 Hz, 1H), 3.98 (dd, *J* = 12.2, 5.7 Hz, 1H), 2.42 (s, 3H); ^13^C-NMR (100 MHz, CDCl_3_): 168.1, 147.5, 139.6, 133.5, 132.2, 131.1, 128.3, 127.5, 126.4, 115.7, 109.7, 77.4, 60.7, 25.4; EIMS (*m*/*z*): 287 [M^+^]; Anal. Calcd. for C_16_H_14_ClNO_2_: C, 66.79; H, 4.90; N, 4.87; Found: C, 67.09; H, 4.99; N, 4.54.


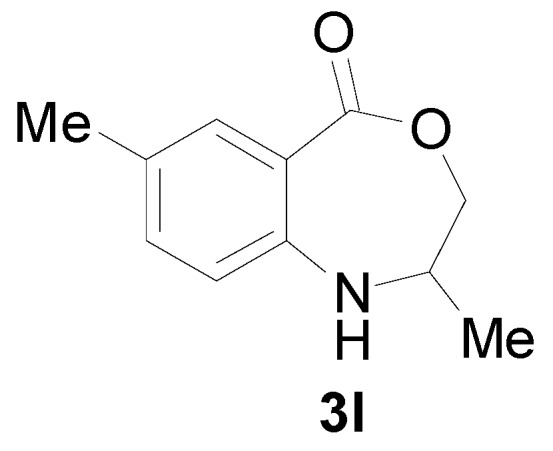


*2,7-Dimethyl-2,3-dihydro-1H-benzo[e][1,4] xazepine-5-one* (**3l**): Yellowish oil. ^1^H-NMR (400 MHz, CDCl_3_): 7.62 (m, 1H), 7.43 (br, 1H), 7.04–7.20 (m, 2H), 4.56 (dd, *J* = 12.2, 8.0 Hz, 1H), 3.93 (dd, *J* = 12.2, 5.4 Hz, 1H), 3.10–3.70 (m, 1H), 2.41 (s, 3H), 1.34 (d, *J* = 7.0 Hz, 3H); ^13^C-NMR (100 MHz, CDCl_3_): 168.3, 147.1, 133.5, 130.9, 126.8, 115.8, 109.2, 77.5, 53.4, 25.3, 18.3; EIMS (*m*/*z*): 191 [M^+^]; Anal. Calcd. for C_11_H_13_NO_2_: C, 69.09; H, 6.85; N, 7.32; Found: C, 69.41; H, 6.55; N, 7.16.


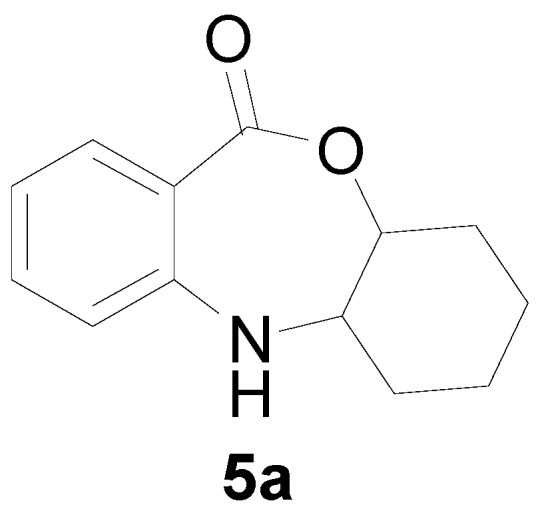


*5a,6,7,8,9,9a-Hexahydro-5H-10-oxa-5-aza-dibenzo[a,d]cyclohepten-11-one* (**5a**): Yellowish oil. ^1^H-NMR (400 MHz, CDCl_3_): 7.60 (m, 1H), 7.48 (br, 1H), 7.02–7.39 (m, 3H), 4.22 (dd, *J* = 11.3, 3.4 Hz, 1H), 3.11 (dd, *J* = 11.3, 3.5 Hz, 1H), 1.61–1.93 (m, 4H), 1.43–1.52 (m, 4H); ^13^C-NMR (100 MHz, CDCl_3_): 168.2, 147.6, 132.6, 130.1, 118.2, 115.9, 108.8, 85.8, 56.1, 28.5, 27.6, 22.9, 21.7; EIMS (*m*/*z*): 217 [M^+^]; Anal. Calcd. for C_13_H_15_NO_2_: C, 71.87; H, 6.96; N, 6.45; Found: C, 71.72; H, 6.66; N, 6.73.


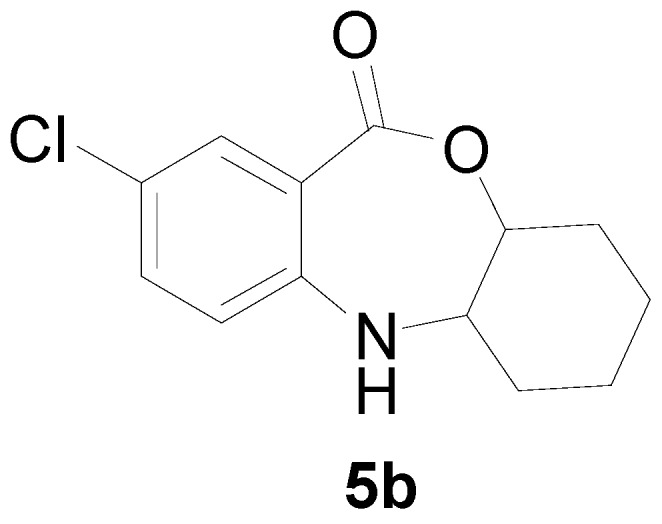


*2-Chloro-5a,6,7,8,9,9a-hexahydro-5H-10-oxa-5-aza-dibenzo[a,d]cyclohepten-11-one* (**5b**): Yellowish oil. ^1^H-NMR (400 MHz, CDCl_3_): 7.62 (m, 1H), 7.49 (br, 1H), 7.05–7.43 (m, 2H), 4.26 (dd, *J* = 11.3, 3.5 Hz, 1H), 3.11 (dd, *J* = 11.3, 3.7 Hz, 1H), 1.62–1.95 (m, 4H), 1.43–1.54 (m, 4H); ^13^C-NMR (100 MHz, CDCl_3_): 168.3, 147.1, 133.1, 130.4, 122.5, 116.1, 108.2, 85.6, 56.5, 28.8, 27.2, 22.7, 21.5; EIMS (*m*/*z*): 251 [M^+^]; Anal. Calcd. for C_13_H_14_ClNO_2_: C, 62.03; H, 5.61; N, 5.56; Found: C, 62.19; H, 5.31; N, 5.34.


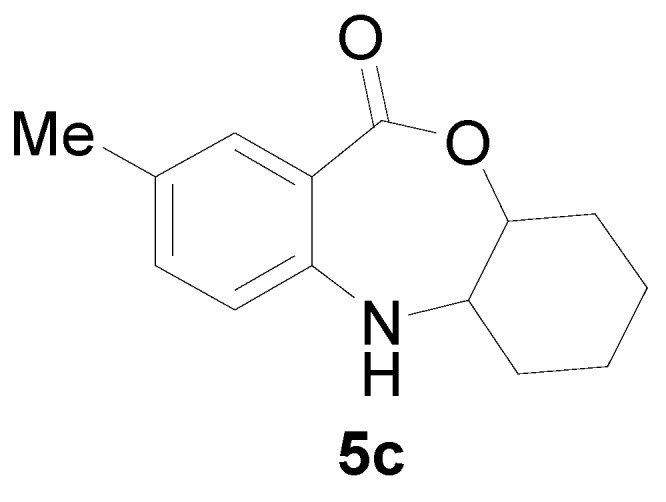


*2-Methyl-5a,6,7,8,9,9a-hexahydro-5H-10-oxa-5-aza-dibenzo[a,d]cyclohepten-11-one* (**5c**): Yellowish oil. ^1^H-NMR (400 MHz, CDCl_3_): 7.58 (m, 1H), 7.46 (1H, br), 7.00–7.35 (m, 2H), 4.20 (dd, *J* = 11.2, 3.2 Hz, 1H), 3.09 (dd, *J* = 11.2, 3.4 Hz, 1H), 2.40 (s, 3H), 1.60–1.91 (m, 4H), 1.42–1.50 (m, 4H); ^13^C-NMR (100 MHz, CDCl_3_): 168.4, 147.3, 133.4, 130.8, 126.1, 116.1, 108.5, 85.4, 56.3, 28.7, 27.8, 22.8, 21.5; EIMS (*m*/*z*): 231 [M^+^]; Anal. Calcd. for C_14_H_17_NO_2_: C, 72.70; H, 7.41; N, 6.06; Found: C, 72.99; H, 7.28; N, 6.48.

## 3. Experimental Section

### 3.1. General Procedure for Preparation of ***L1***–***L6***

Dimethylformamide dimethyl acetal (DMF-DMA) (10 mmol, 1.19 g) and 1-(1-hydroxy-1*H*-inden-2-yl)-ethanone (10 mmol, 1.74 g) were dissolved in *p*-xylene (5 mL). Additionally, the mixture was refluxed during a period of 5–12 h, during which time a yellow precipitate formed. The precipitate was filtered out and washed with petroleum ether three times. The solid was vacuum-dried, and 1.89 g (yield 94%) of a yellow solid were obtained, **L1** 2-(2-dimethylamino-vinyl)-1*H*-inden-1-ol. ^1^H-NMR (400 MHz, CDCl_3_): δ 7.23 (m, 2H), 7.17–7.07 (t, *J* = 8.0 Hz, 2H), 7.01–6.90 (t, *J* = 7.8 Hz, 1H), 6.60 (s, 1H), 6.07–6.05 (d, *J* = 12 Hz, 1H), 2.47 (s, 3H), 2.42 (s, 3H); ^13^C-NMR (100 MHz, CDCl_3_): δ 146.1, 141.2, 133.8, 130.2, 127.9, 126.9, 123.2,121.2, 120.6, 104.1, 75.4, 46.1, 38.6.

### 3.2. 2-Phenyl-2,3-dihydro-1H-benzo[e][1,4]oxazepin-5-one *(**3a**)*

A mixture of phenylamine **1a** (0.5 mmol, 46.5 mg), (1-chloro-vinyl)-benzene **2a** (0.6 mmol, 83.4 mg), CuI (10 mol %, 9.5 mg), **L1** (10 mol %, 20.1 mg) and Cs_2_CO_3_ (2 equiv., 325.8 mg) in DMSO (4 mL) was stirred in CO_2_ at 100 °C for 10 h. After completion of the reaction, the mixture was quenched with saturated salt water (10 mL); the solution was extracted with ethyl acetate (3 × 10 mL). The organic layers were combined and dried over sodium sulfate. The pure product was obtained by flash column chromatography on silica gel to afford **3a** 96.8 mg in 81% yield. The spectroscopic data of all of the products are represented below. Yellowish oil. ^1^H-NMR (400 MHz, CDCl_3_): 7.63 (m, 1H), 7.43 (br, 1H), 7.08–7.43 (m, 8H), 5.07 (dd, *J* = 8.0, 5.7 Hz, 1H), 4.08 (dd, *J* = 12.3, 8.0 Hz, 1H), 3.96 (dd, *J* = 12.3, 5.6 Hz, 1H); ^13^C-NMR (100 MHz, CDCl_3_): 168.3, 147.7, 139.1, 132.9, 130.3, 128.6, 127.5, 126.6, 117.8, 116.4, 109.1, 77.6, 60.2; EIMS (*m*/*z*): 239 [M^+^]; Anal. Calcd. for C_15_H_13_NO_2_: C, 75.30; H, 5.48; N, 5.85; Found: C, 75.62; H, 5.13; N, 5.68.

## 4. Conclusions

In conclusion, we have found a green protocol for the synthesis of benzo-1,4-oxazepine derivatives involving tandem transformation of C-N coupling/C-H carbonylation. The method was economically viable and relevant to green chemistry.

## Figures and Tables

**Table 1:**
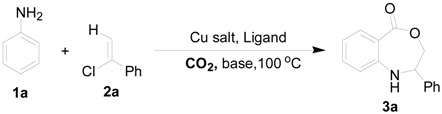
Optimization of the reaction conditions ^a^.

Entry	Ligand	Cu Salt	Base	Yield (%) ^b^
1	**L1**	Cu(OAc)_2_	Cs_2_CO_3_	8
2	**L1**	CuSO_4_	Cs_2_CO_3_	0
3	**L1**	CuBr	Cs_2_CO_3_	23
4	**L1**	CuBr_2_	Cs_2_CO_3_	19
5	**L1**	CuI	Cs_2_CO_3_	81
6	**L2**	CuI	Cs_2_CO_3_	29
7	**L3**	CuI	Cs_2_CO_3_	36
8	**L4**	CuI	Cs_2_CO_3_	47
9	**L5**	CuI	Cs_2_CO_3_	16
10	**L6**	CuI	Cs_2_CO_3_	38
11	**L1**	CuI	K_2_CO_3_	42
12	**L1**	CuI	K_3_PO_4_	0
13	**L1**	CuI	Cs_2_CO_3_	61 ^c^
14	**L1**	CuI	Cs_2_CO_3_	69 ^d^
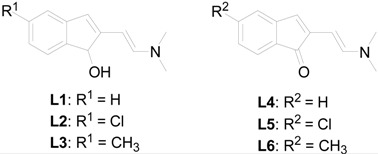				

^a^ Unless otherwise noted, reactions conditions were **1a** (0.5 mmol), **2a** (0.6 mmol), Cu salt (10 mol %), ligand (10 mol %), base (2 eq.), DMSO (4 mL) reacted in CO_2_ at 100 °C for 12 h; ^b^ isolated yield; ^c^ reaction under 90 °C; ^d^ reaction under 110 °C.

**Table 2:**
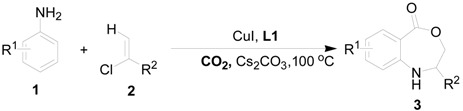
Synthesis of benzo-1,4-oxazepin-5-one **3** ^a^.

Entry	R^1^	R^2^	Product 3	Yield (%) ^b^
1	H	Ph	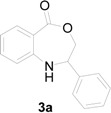	81
2	H	4-CH_3_C_6_H_4_	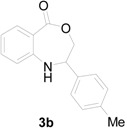	78
3	H	4-ClC_6_H_4_	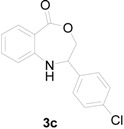	85
4	H	CH_3_	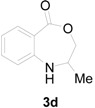	74
5	4-Cl	Ph	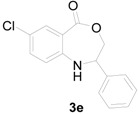	79
6	4-Cl	4-CH_3_C_6_H_4_	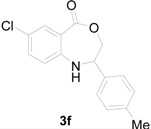	76
7	4-Cl	4-ClC_6_H_4_	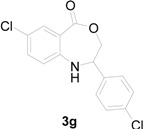	86
8	4-Cl	CH_3_	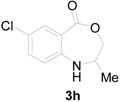	84
9	4-CH_3_	Ph	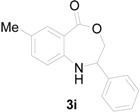	76
10	4-CH_3_	4-CH_3_C_6_H_4_	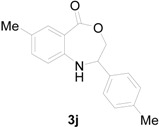	75
11	4-CH_3_	4-ClC_6_H_4_	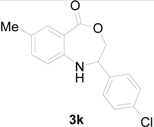	82
12	4-CH_3_	CH_3_	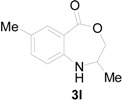	72

^a^ Reactions conditions were **1** (0.5 mmol), **2** (0.6 mmol), CuI (10 mol %), **L1** (10 mol %), Cs_2_CO_3_ (2 equiv.), DMSO (4 mL) at 100 °C reacted in CO_2_ for 10 h; ^b^ isolated yield.

**Table 3:**
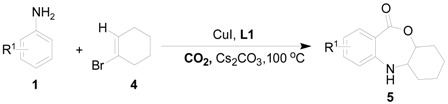
Synthesis of benzo-1,4-oxazepin-5-one **5** ^a^.

Entry	R^1^	Product 5	Yield (%) ^b^
1	H	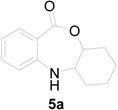	78
2	4-Cl	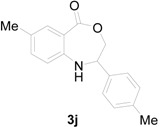	84
3	4-CH_3_	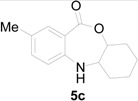	75

^a^ Reactions conditions were **1** (0.5 mmol), **2** (0.6 mmol), CuI (10 mol %), **L1** (10 mol %), Cs_2_CO_3_ (2 equiv.), DMSO (4 mL) at 100 °C reacted in CO_2_ for 10 h; ^b^ isolated yield.

## Supplementary Materials

Supplementary materials can be accessed at: http://www.mdpi.com/1420-3049/22/1/53 /s1.
