# Supplementary material for: Facile Synthesis for Benzo-1,4-Oxazepine Derivatives by Tandem Transformation of C-N Coupling/C-H Carbonylation
Source: Molecules. 2016 Dec 30;22(1):53. doi: 10.3390/molecules22010053 (PMC6155786; doi:10.3390/molecules22010053)
Supplement: Supplementary file 1 [file molecules-22-00053-s001.pdf]

Supplementary Materials: Facile Synthesis for Benzo-1,4-Oxazepine Derivatives by Tandem Transformation of C-N Coupling/C-H Carbonylation

Xiaojia Zhao, Jiong Zhang, Zeqin Zheng and Runsheng Xu

1. Experimental Details

1.1. General Information

All reagents used in the experiments were obtained from commercial sources and used without further purification. Unless otherwise noted, all reactions were carried out at CO_2 atmosphere. Thin layer chromatography (TLC) employed glass 0.25-mm silica gel plates. All NMR spectra were recorded on a BrukerAvance II (400 MHz) spectrometer for ^{1}H-NMR (400 MHz) and ^{13}C-NMR (100 MHz) in $CDCl_3$ using TMS as the internal reference. The ^{1}H-NMR spectra were reported in delta (δ) units, parts per million (ppm) downfield from the internal standard. Melting points were tested by the XT-4 apparatus without correcting the temperature or cited from the literature when applicable. The EIMS of new products were tested on the Agilent 6210 LC/MS equipped with an electrospray source.

1.2. General Procedure for Preparation of **L1–L6**

Dimethylformamide dimethylacetal (DMF-DMA) (10 mmol, 1.19 g) and 1-(1-hydroxy-1*H*-inden-2-yl)-ethanone (10 mmol, 1.74 g) were dissolved in *p*-xylene (5 mL). Additionally, the mixture was refluxed during a period of 5–12 h, during which time a yellow precipitate formed. The precipitate was filtered out and washed with petroleum ether three times. The solid was vacuum-dried, and 1.89 g (yield 94%) of a yellow solid wereobtained, **L1** 2-(2-dimethylamino-vinyl)-1*H*-inden-1-ol. ^{1}H-NMR (400 MHz, $CDCl_3$): δ 7.23 (m, 2H), 7.17–7.07 (t, *J* = 8.0 Hz, 2H), 7.01–6.90 (t, *J*= 7.8 Hz, 1H), 6.60 (s, 1H), 6.07–6.05 (d, *J* = 12 Hz, 1H), 2.47 (s, 3H), 2.42 (s, 3H); ^{13}C-NMR (100 MHz, $CDCl_3$): δ 146.1, 141.2, 133.8, 130.2, 127.9, 126.9, 123.2,121.2, 120.6, 104.1, 75.4, 46.1, 38.6.

2. Spectrums

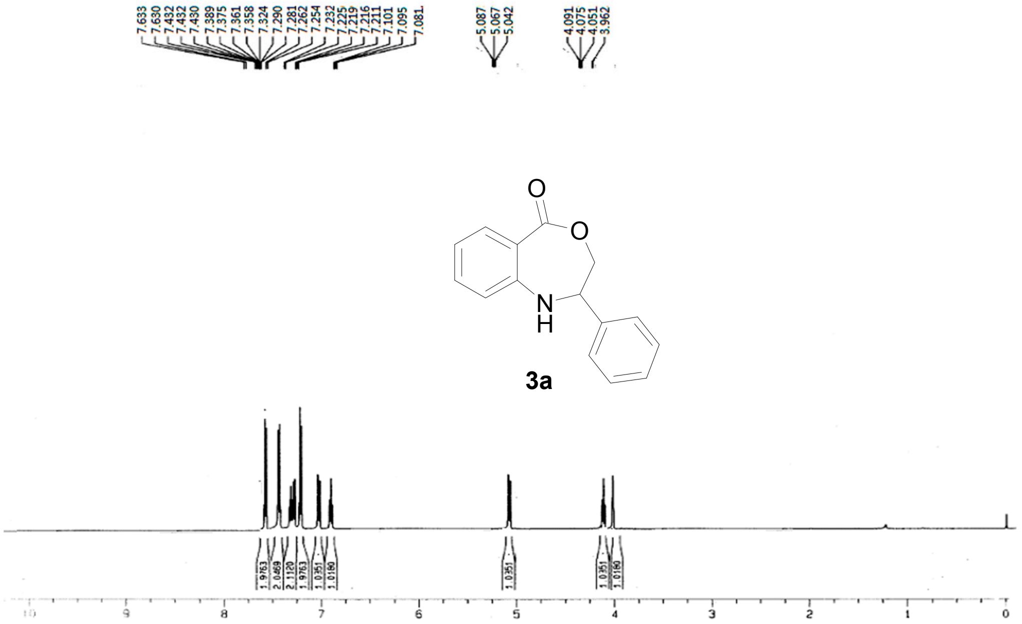

Figure S1. ^{1}H-NMR **3a**.

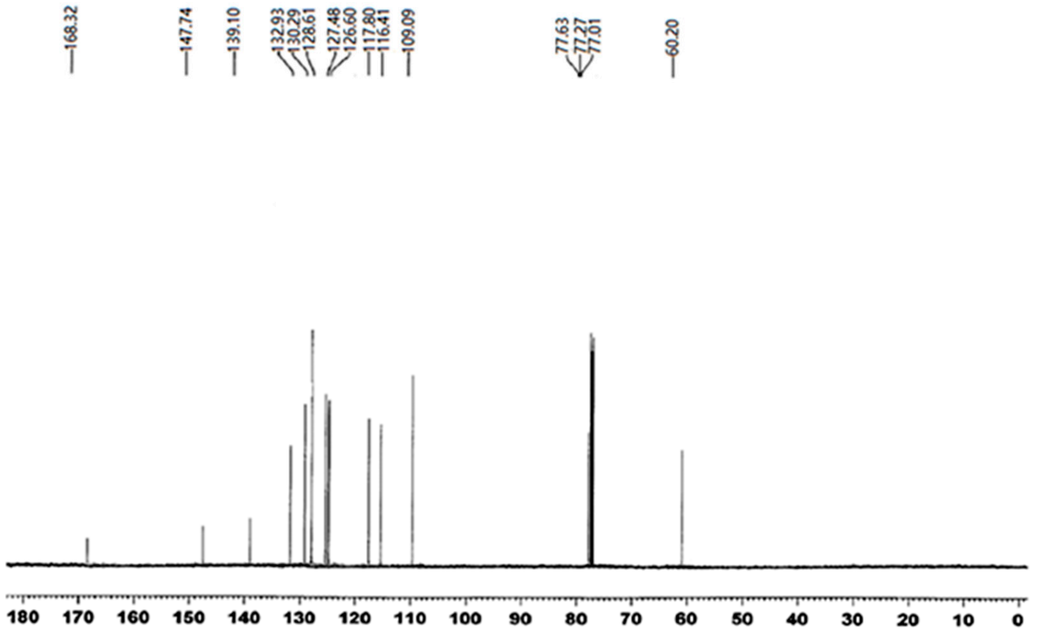

Figure S2. ^{13}C-NMR **3a**.

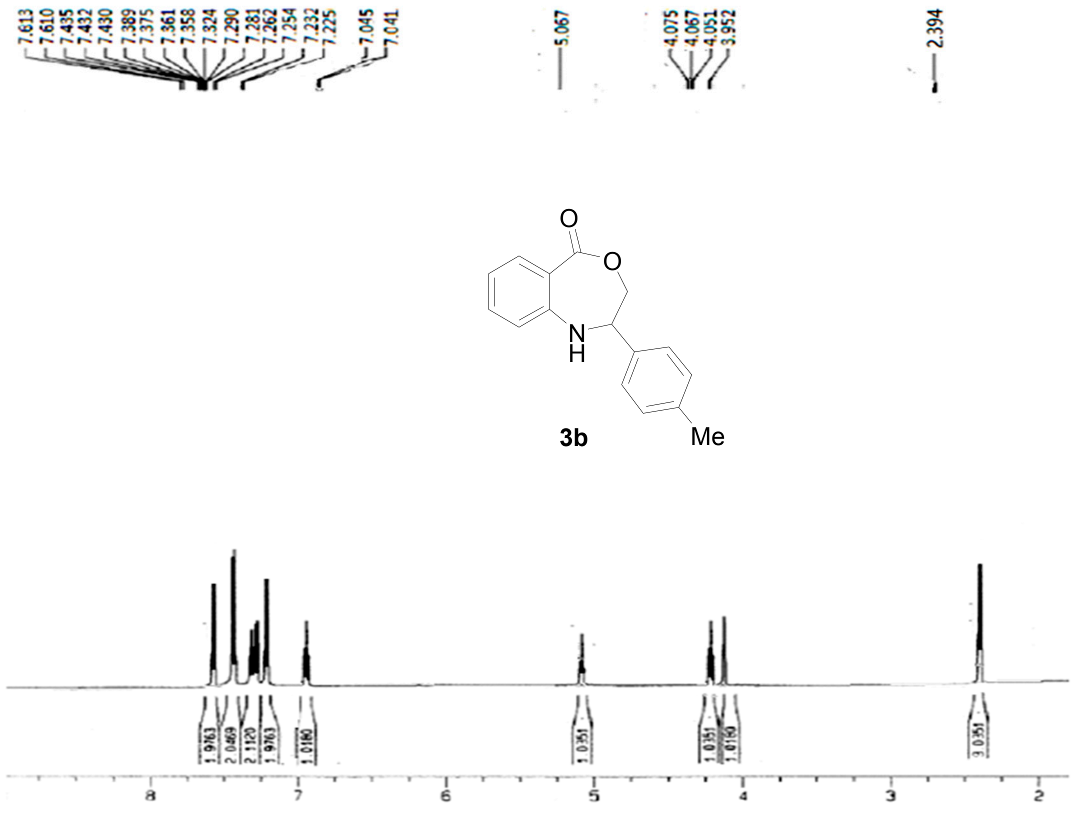

Figure S3. ^{1}H-NMR **3b**.

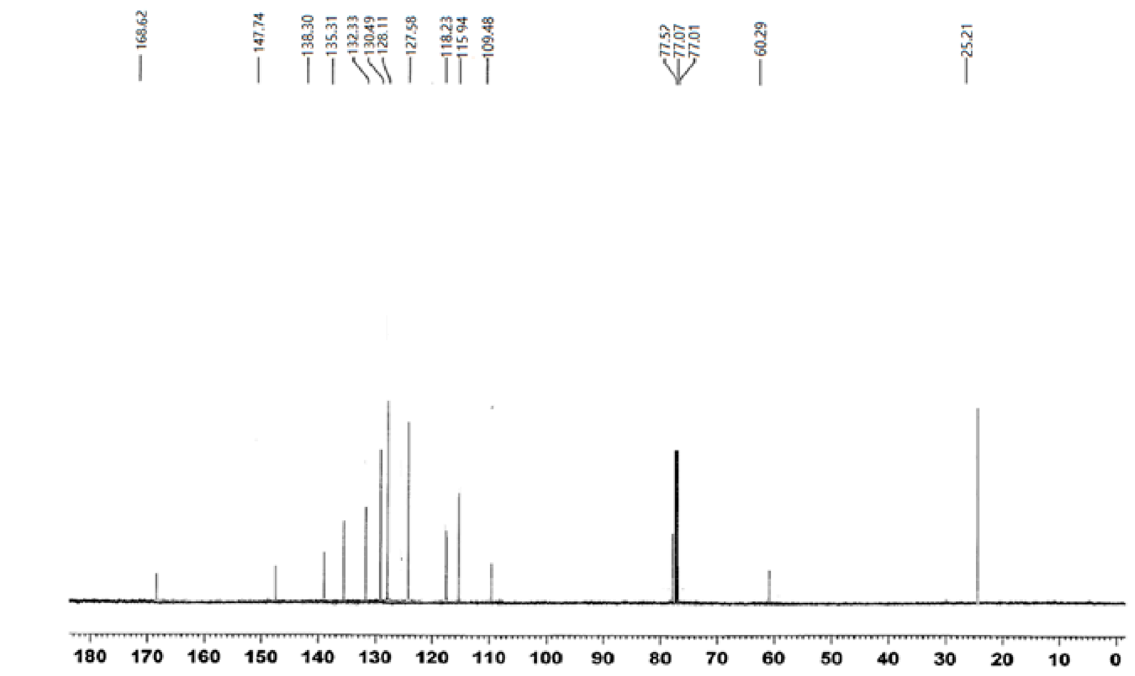

Figure S4. ^{13}C-NMR **3b**.

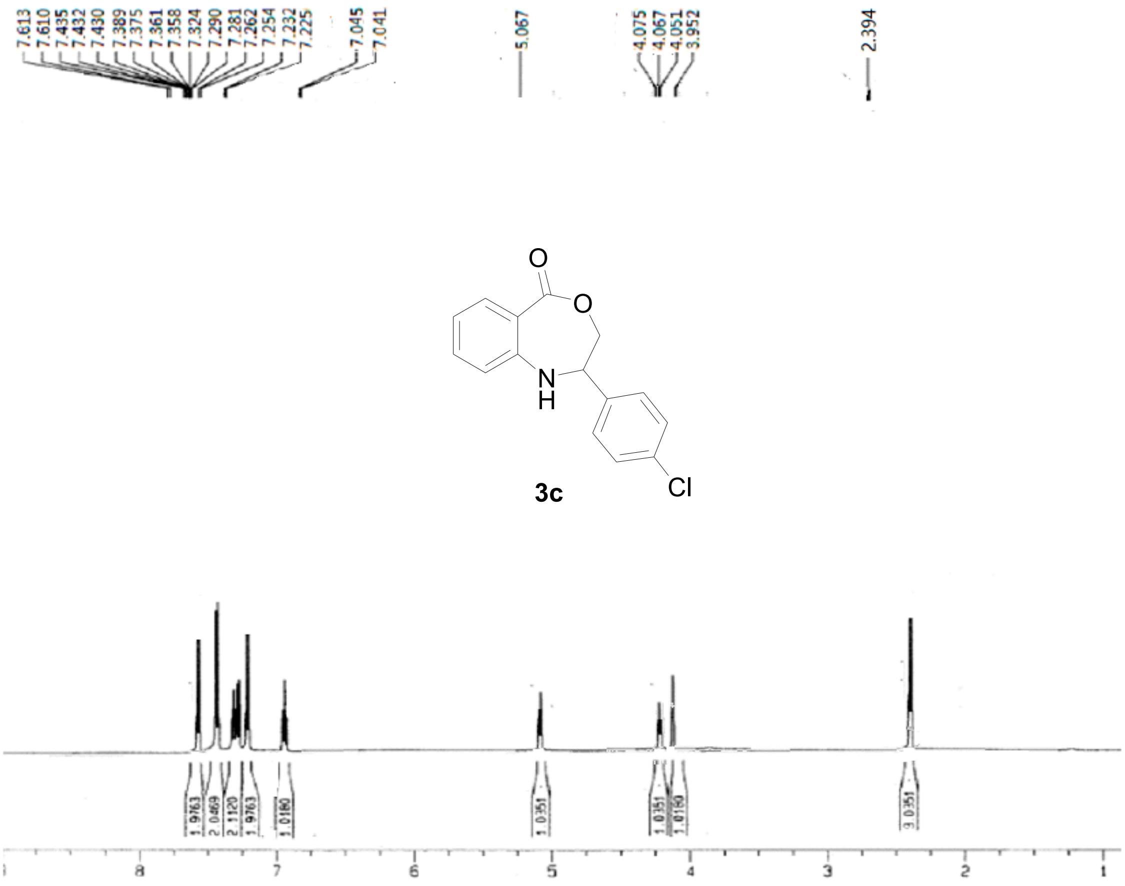

Figure S5. ^{1}H-NMR **3c**.

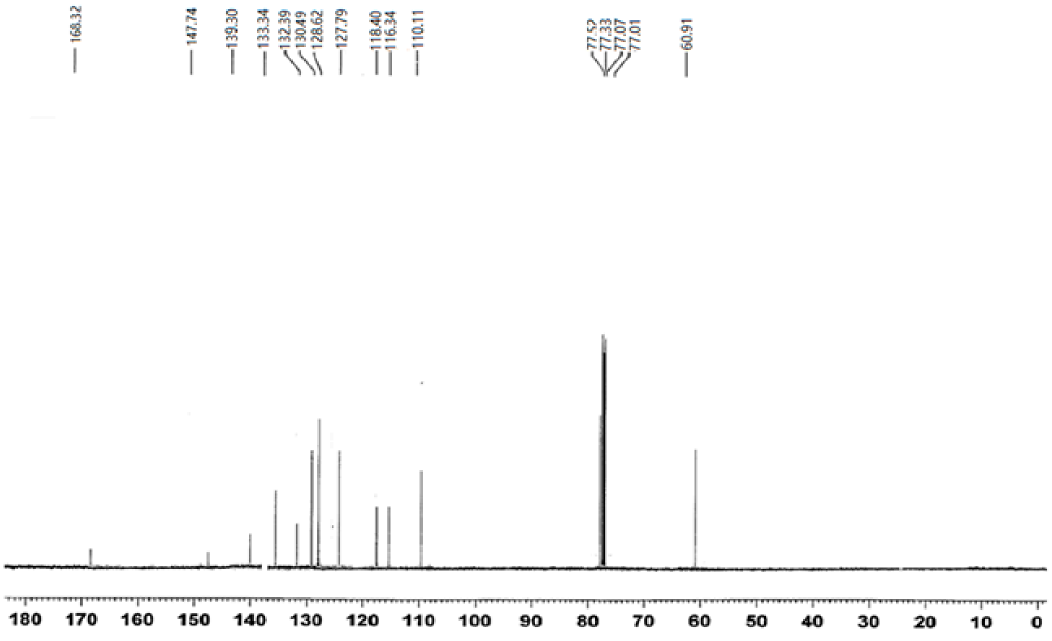

Figure S6. ^{13}C-NMR **3c**.

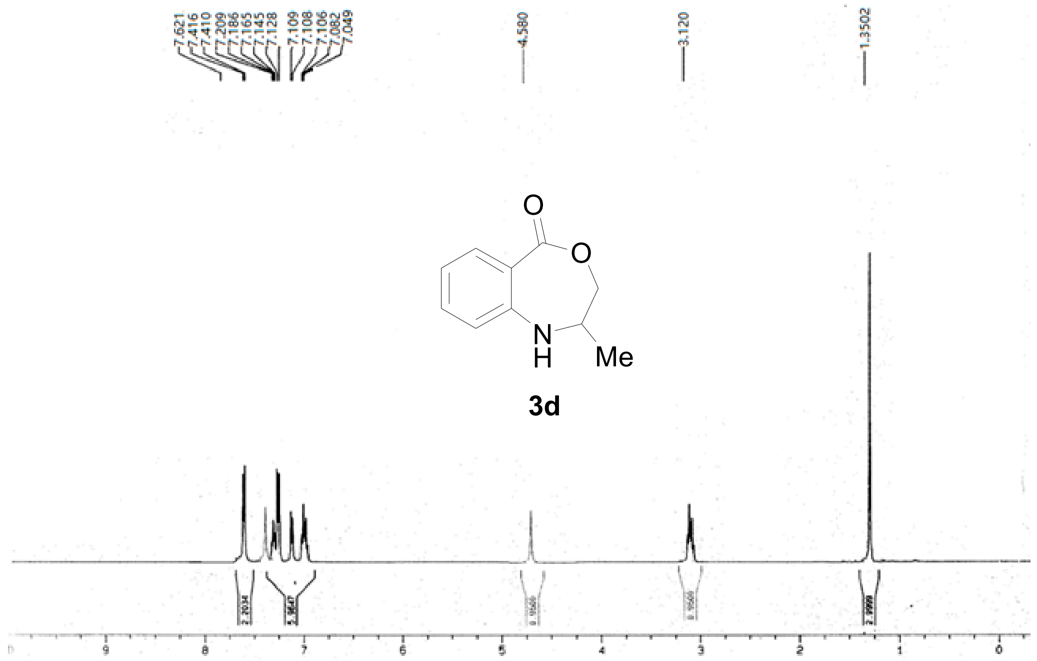

Figure S7. ^{1}H-NMR **3d**.

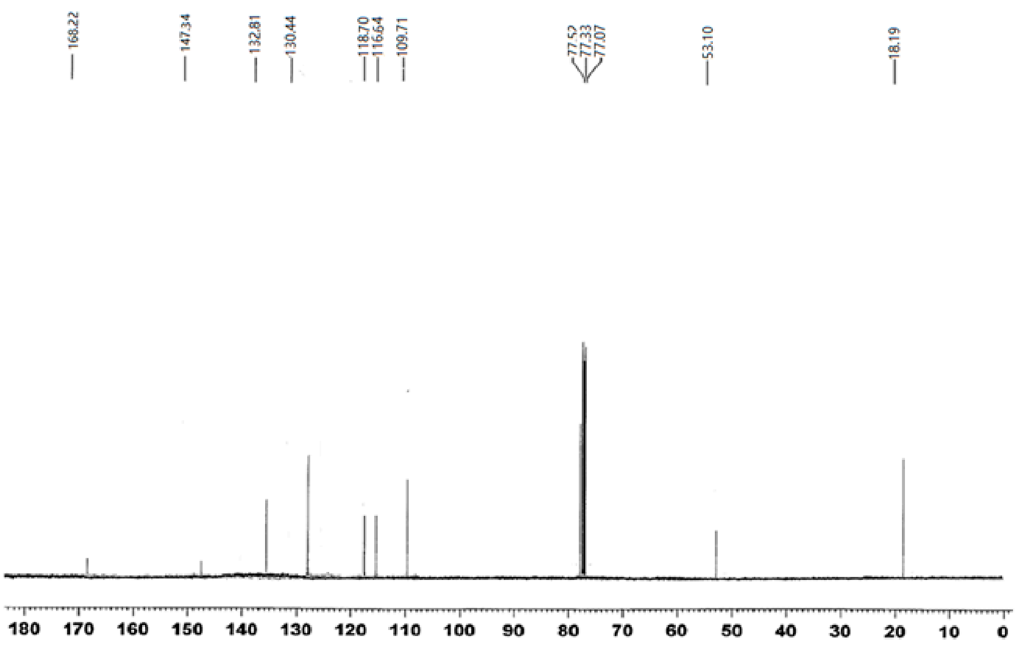

Figure S8. ^{13}C-NMR **3d**.

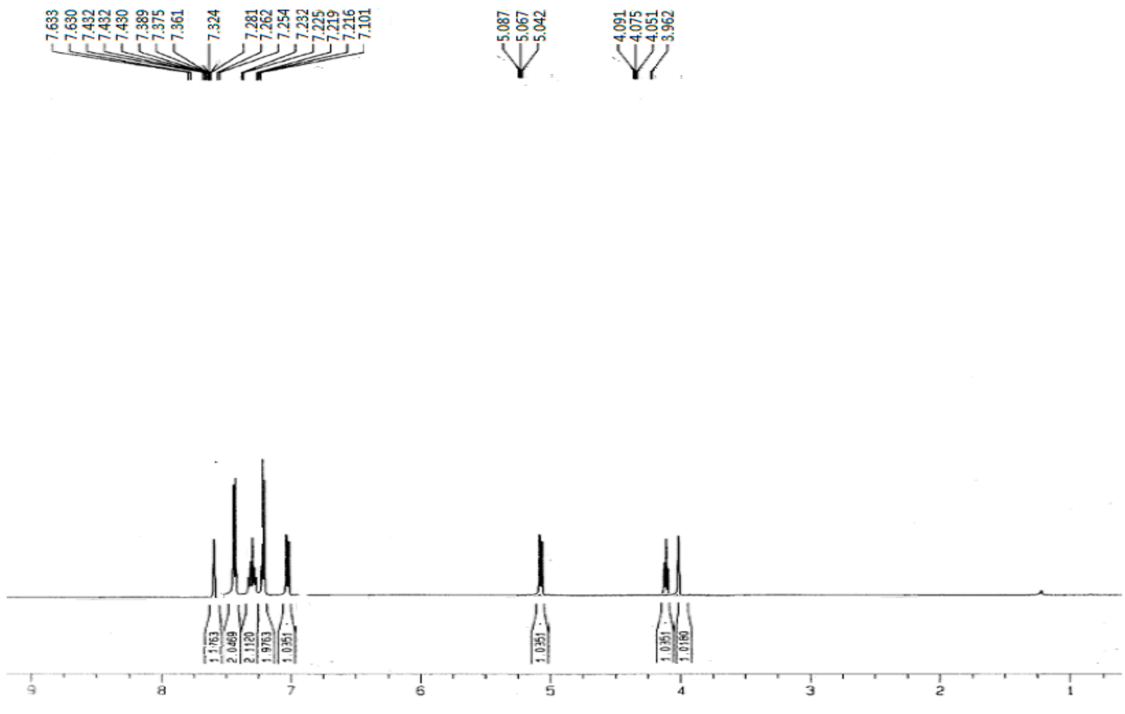

Figure S9. ^{1}H-NMR **3e**.

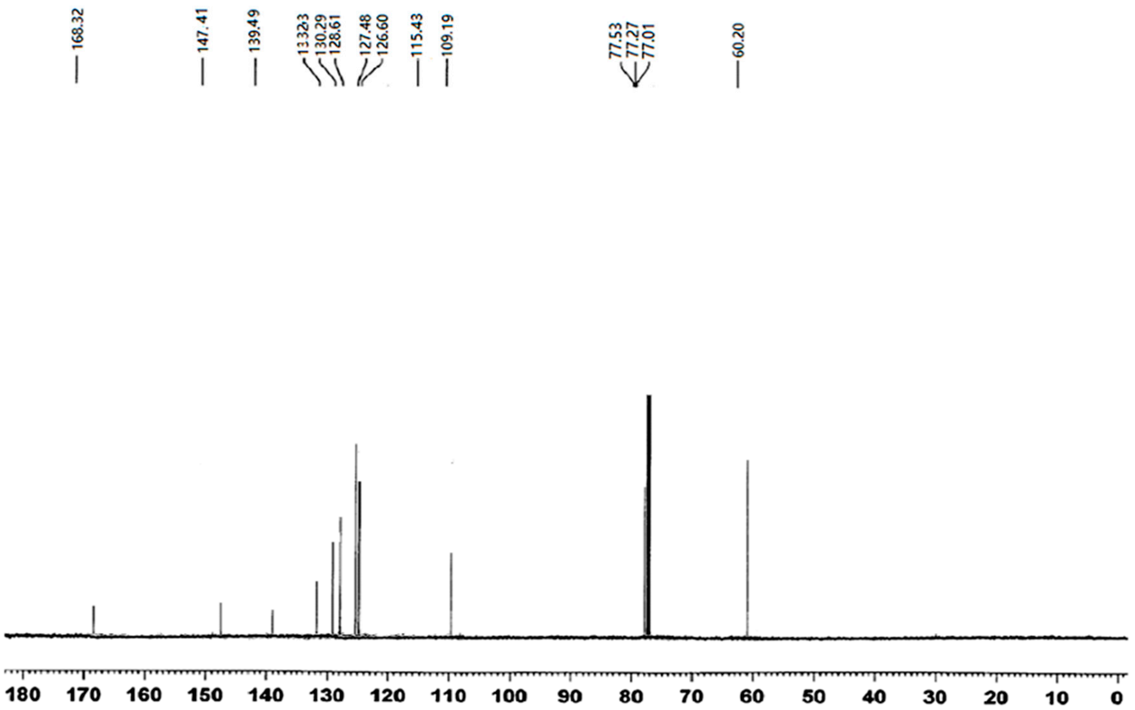

Figure S10. ^{13}C-NMR **3e**.

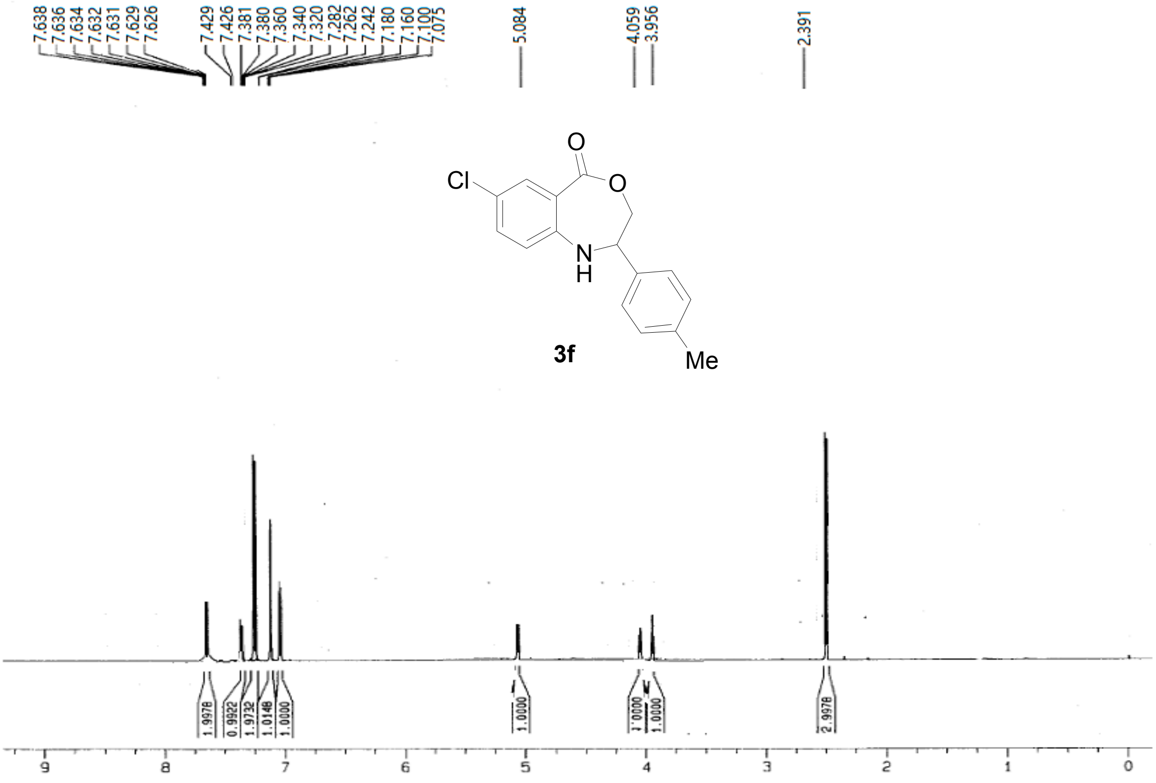

Figure S11. ^{1}H-NMR **3f**.

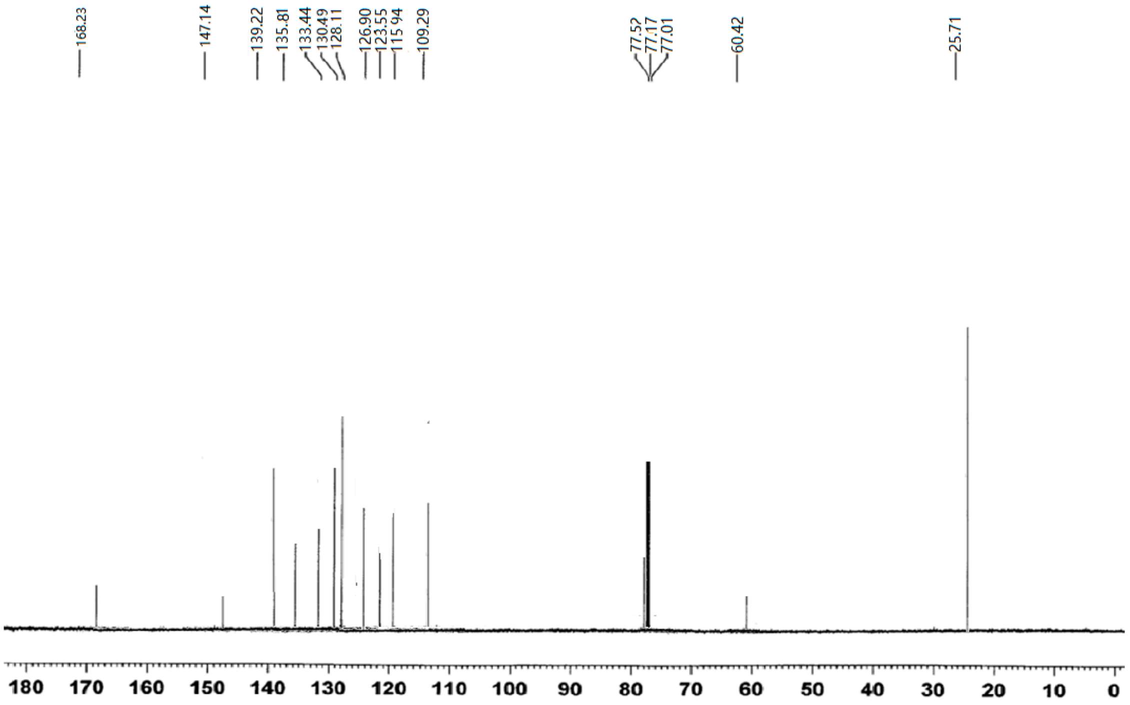

Figure S12. ^{13}C-NMR **3f**.

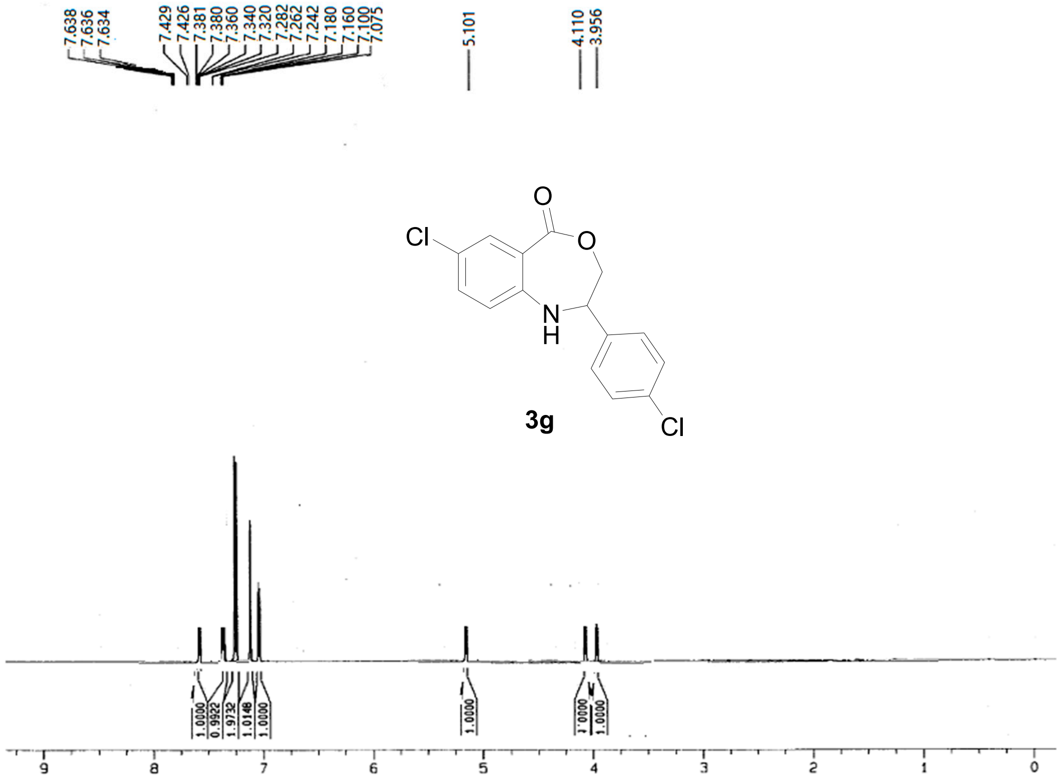

Figure S13. ^{1}H-NMR **3g**.

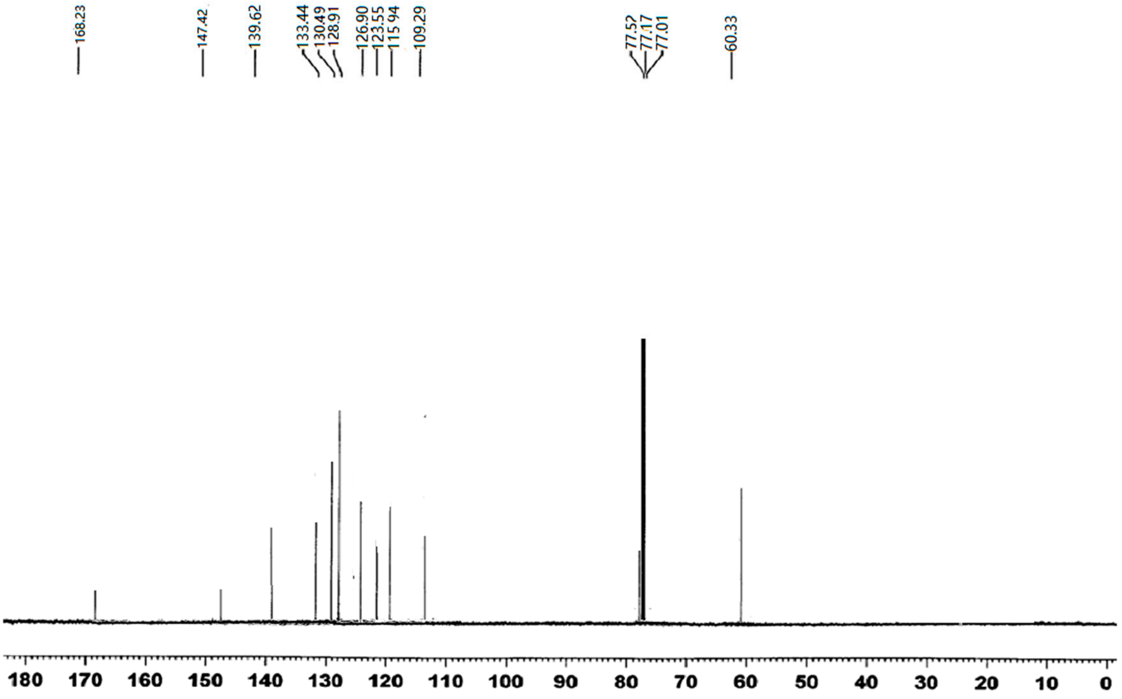

Figure S14. ^{13}C-NMR **3g**.

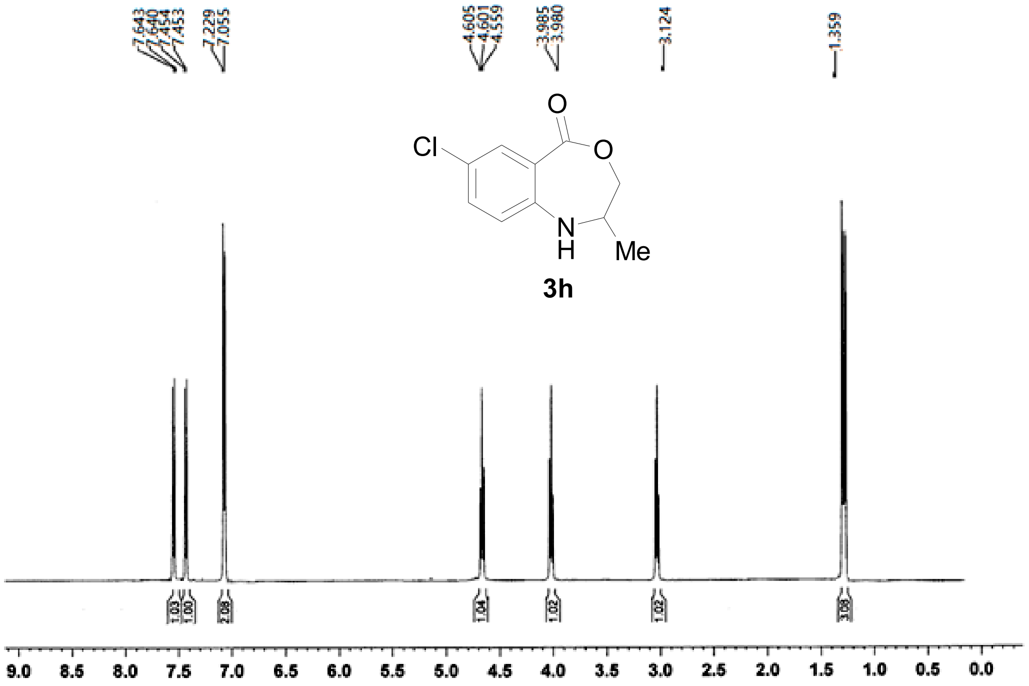

Figure S15. ^{1}H-NMR **3h**.

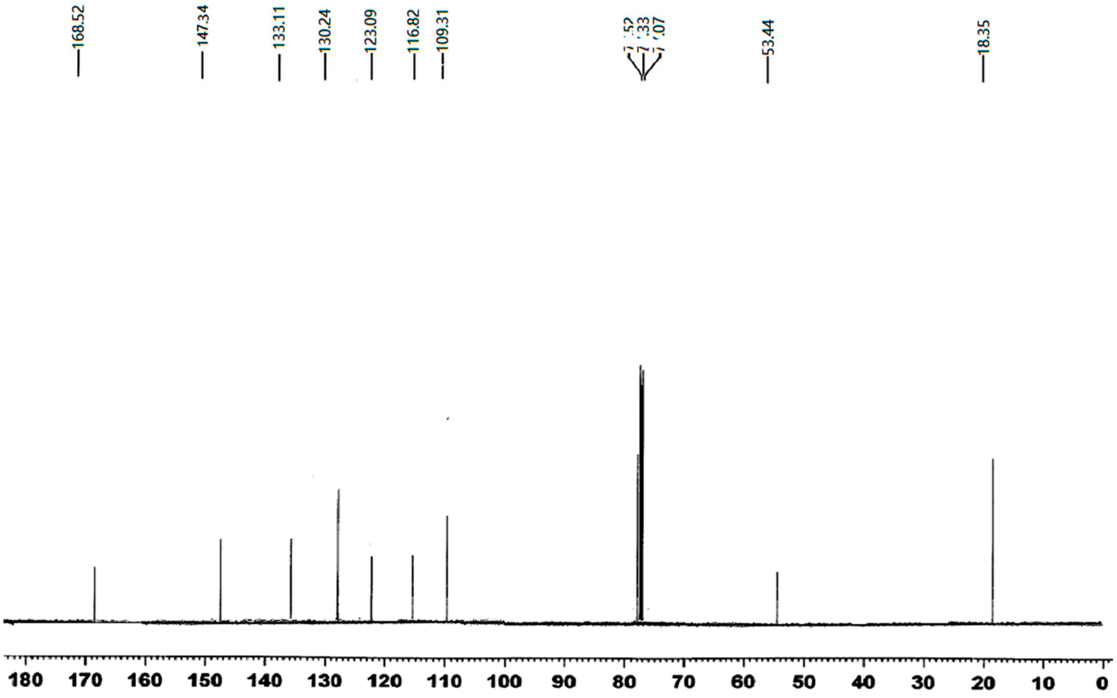

Figure S16. ^{13}C-NMR **3h**.

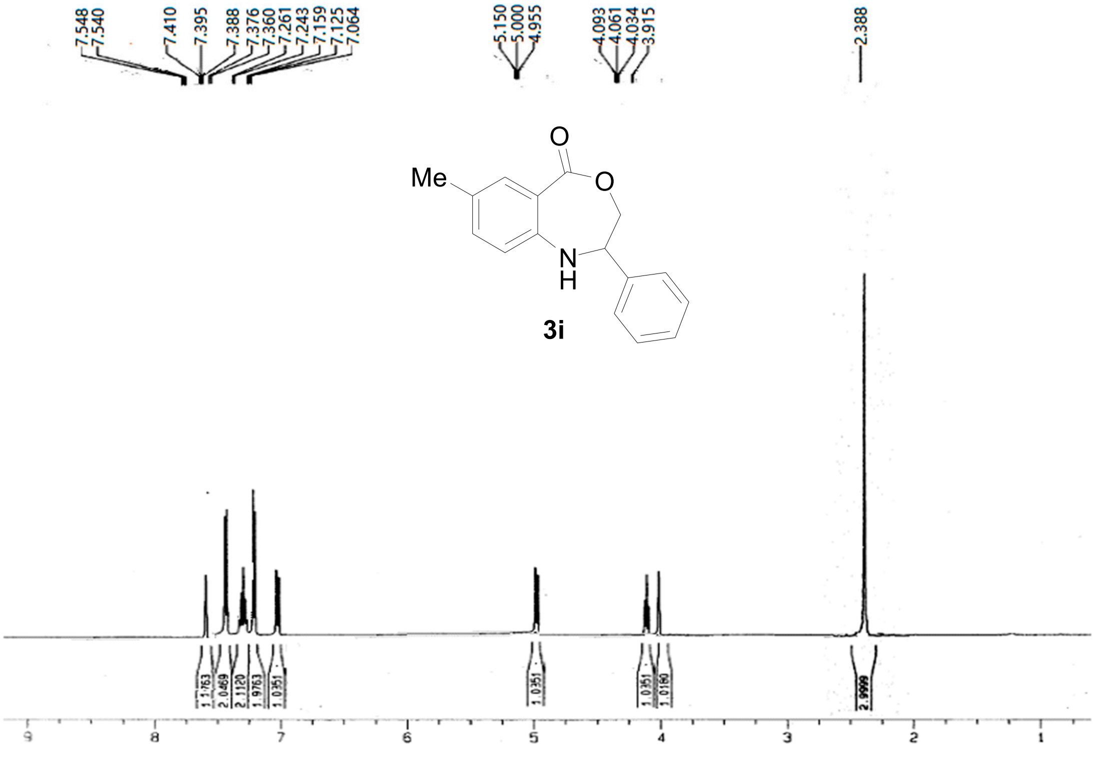

Figure S17. ^{1}H-NMR **3i**.

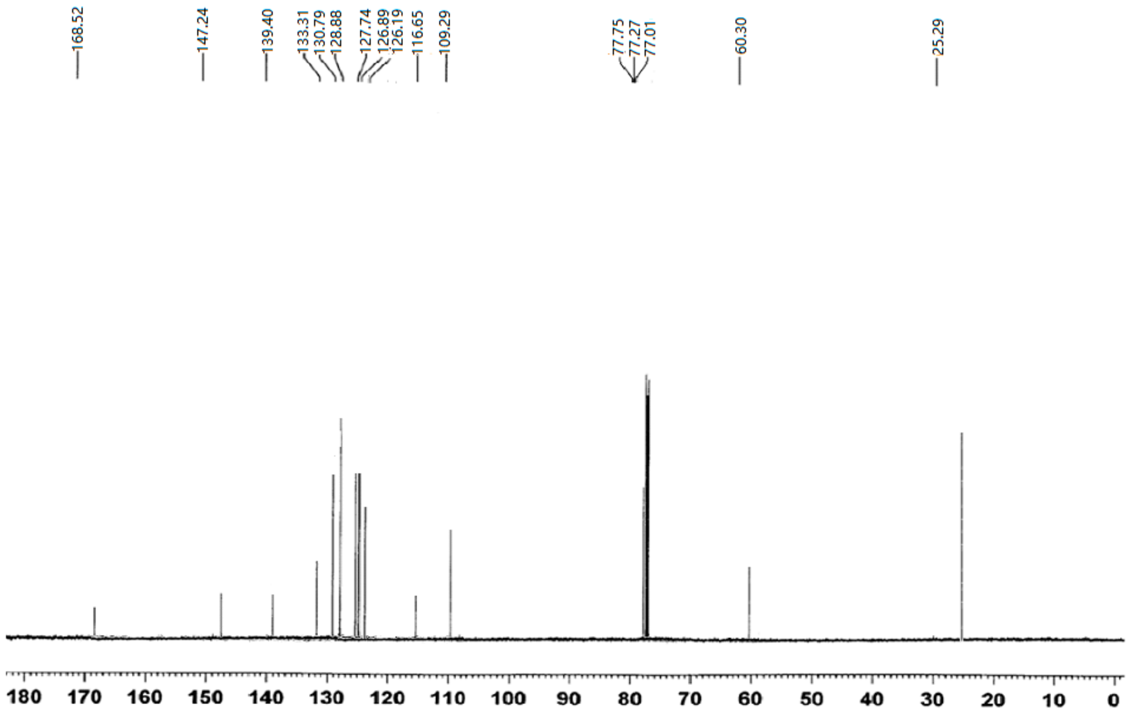

Figure S8. 13C-NMR **3i**.

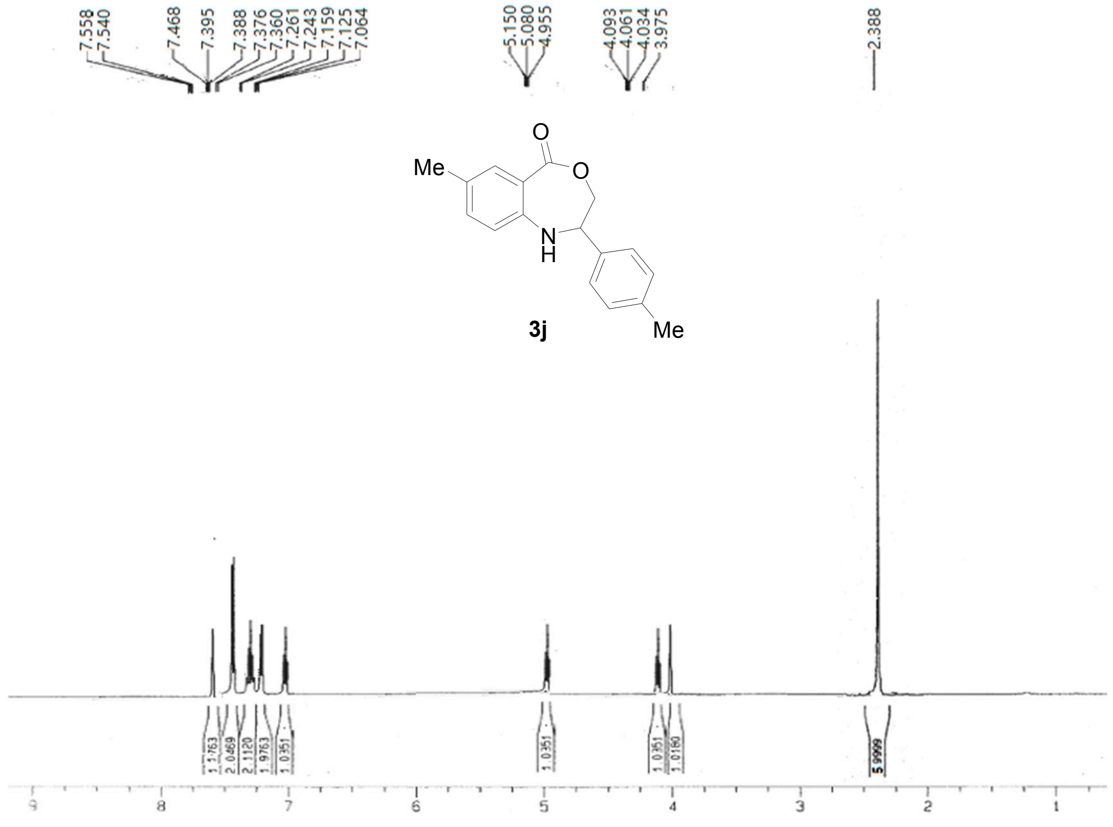

Figure S19. ^{1}H-NMR **3j**.

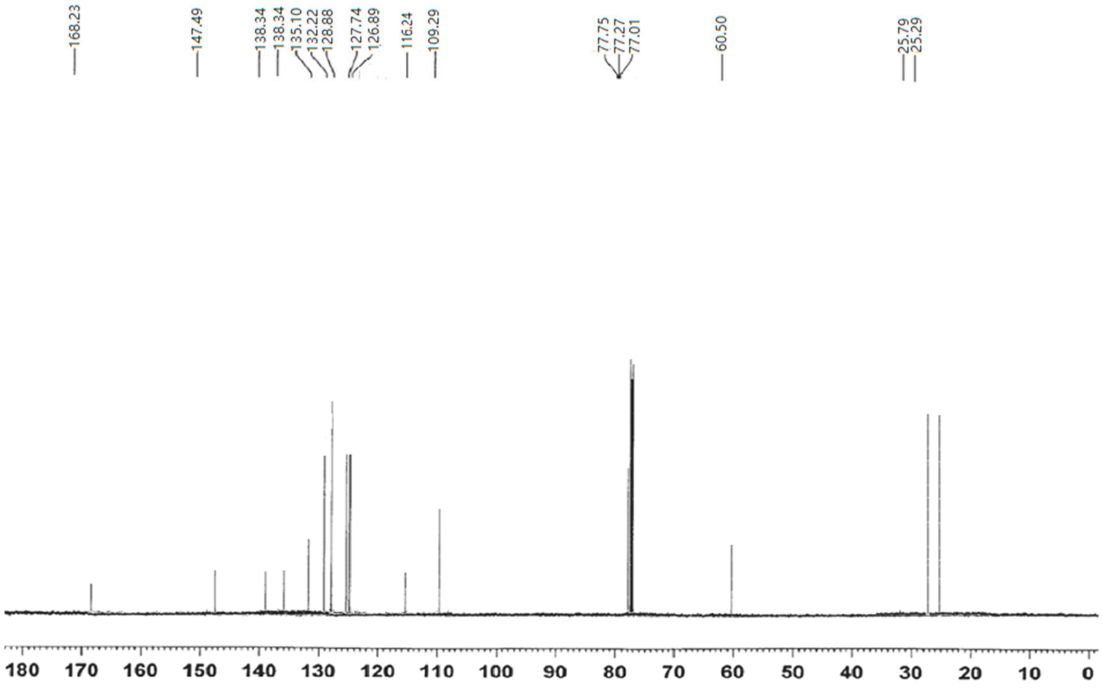

Figure S20. ^{13}C-NMR **3j**.

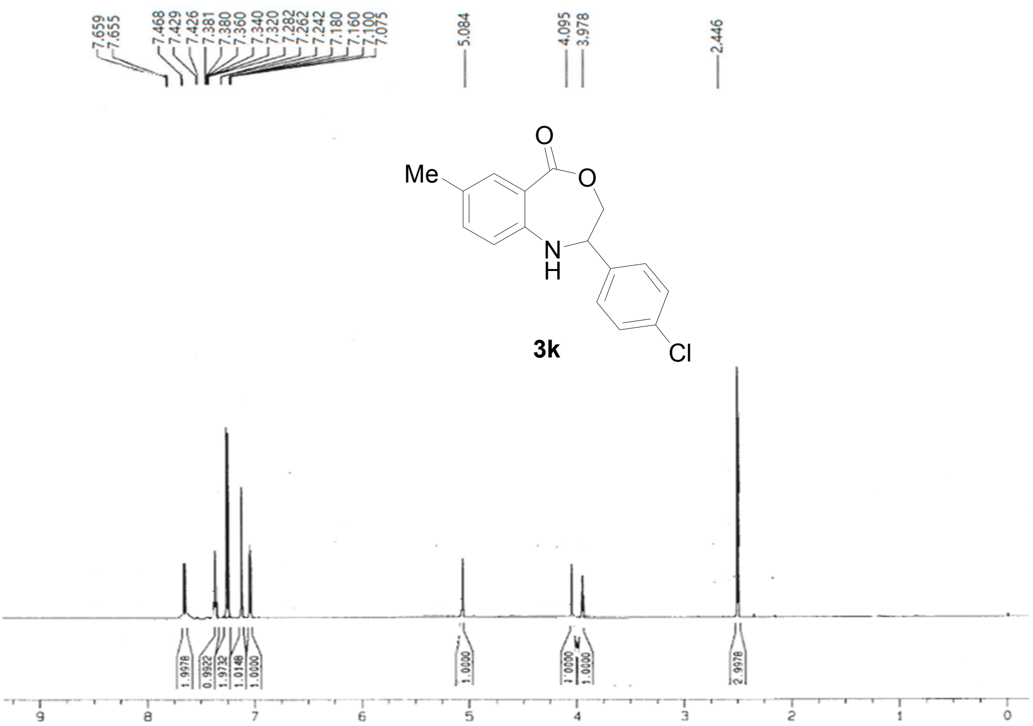

Figure S21. ^{1}H-NMR **3k**.

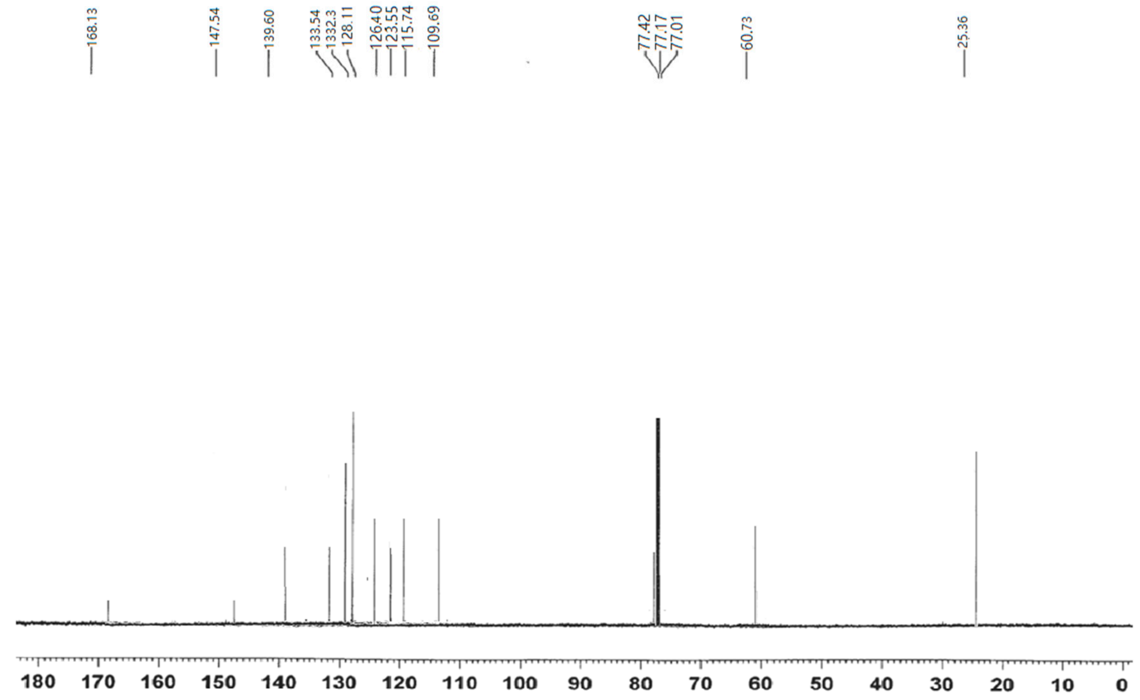

Figure S22. ^{13}C-NMR **3k**.

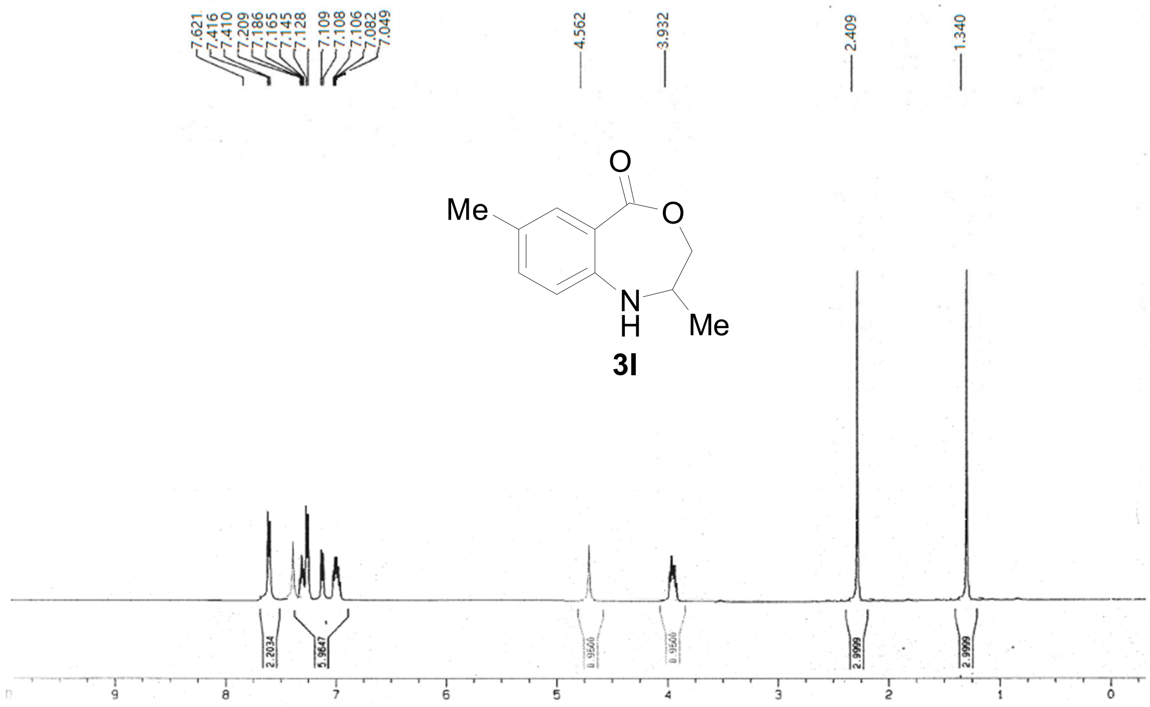

Figure S23. ^{1}H-NMR **3l**.

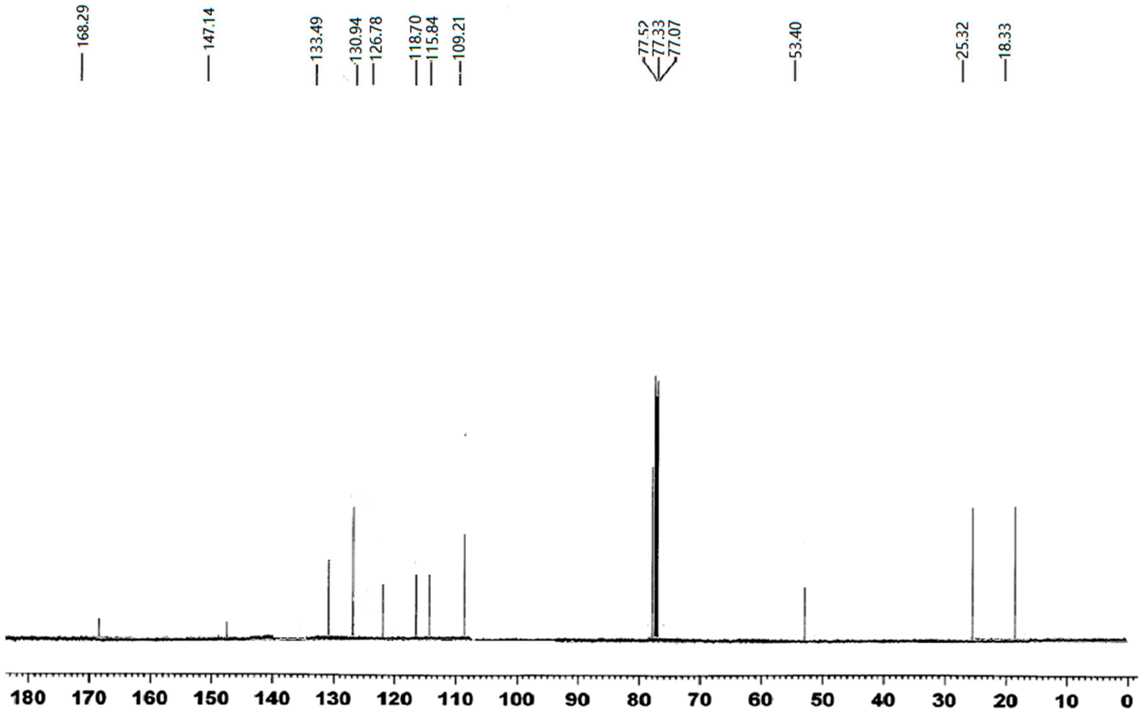

Figure S24. ^{13}C-NMR **3l**.

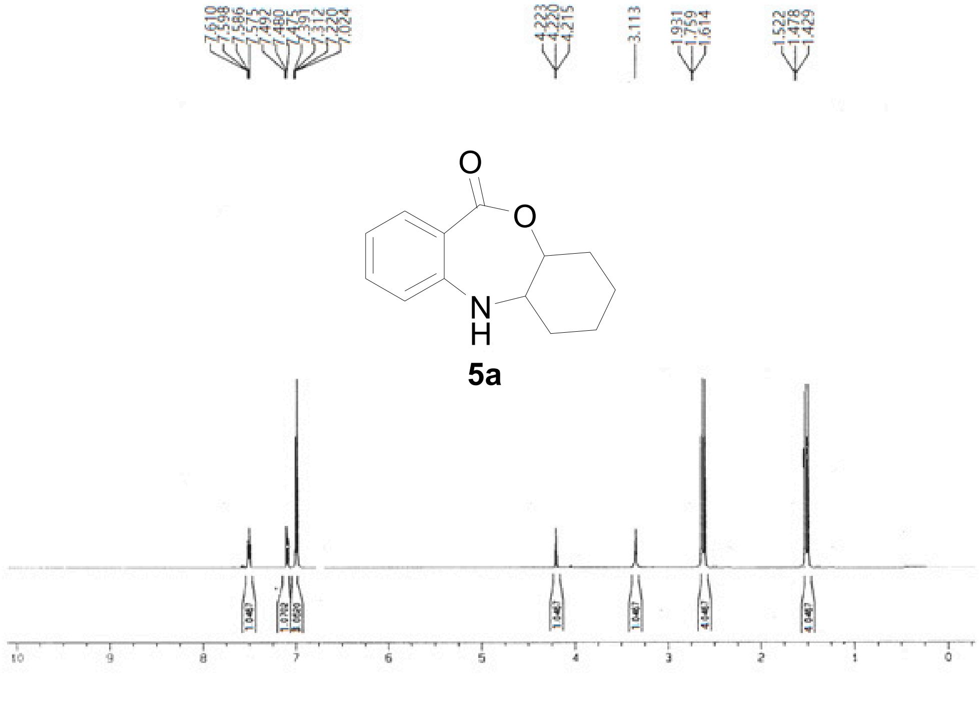

Figure S25. ^{1}H-NMR **5a**.

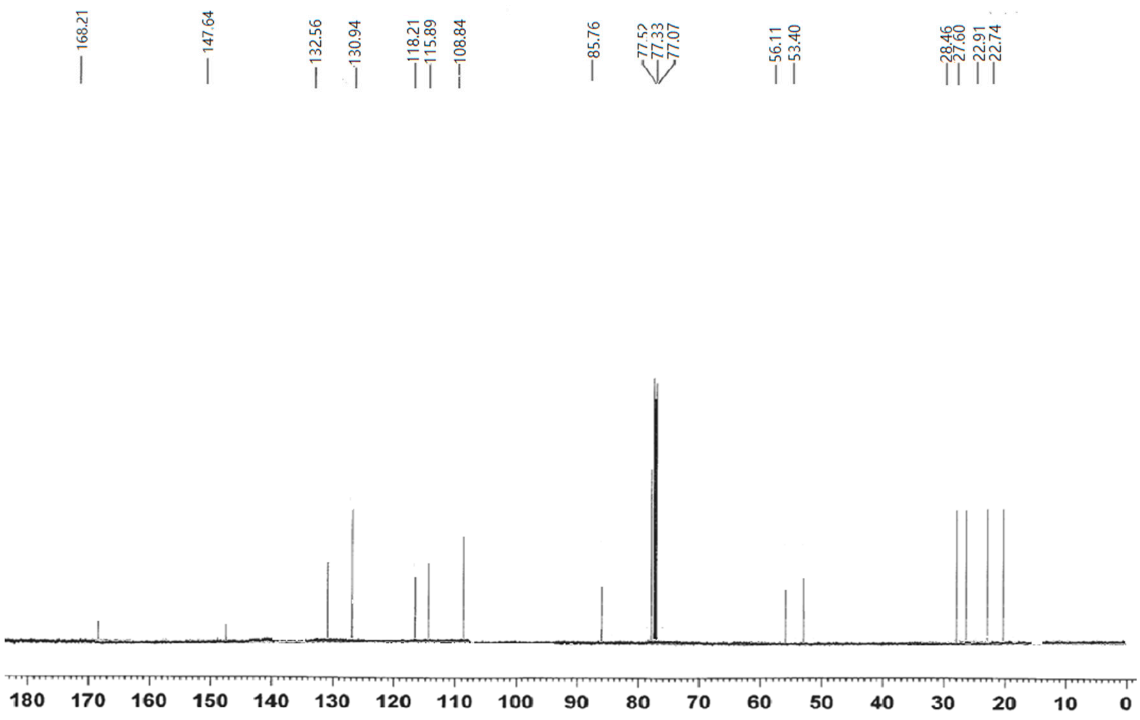

Figure S26. ^{13}C-NMR **5a**.

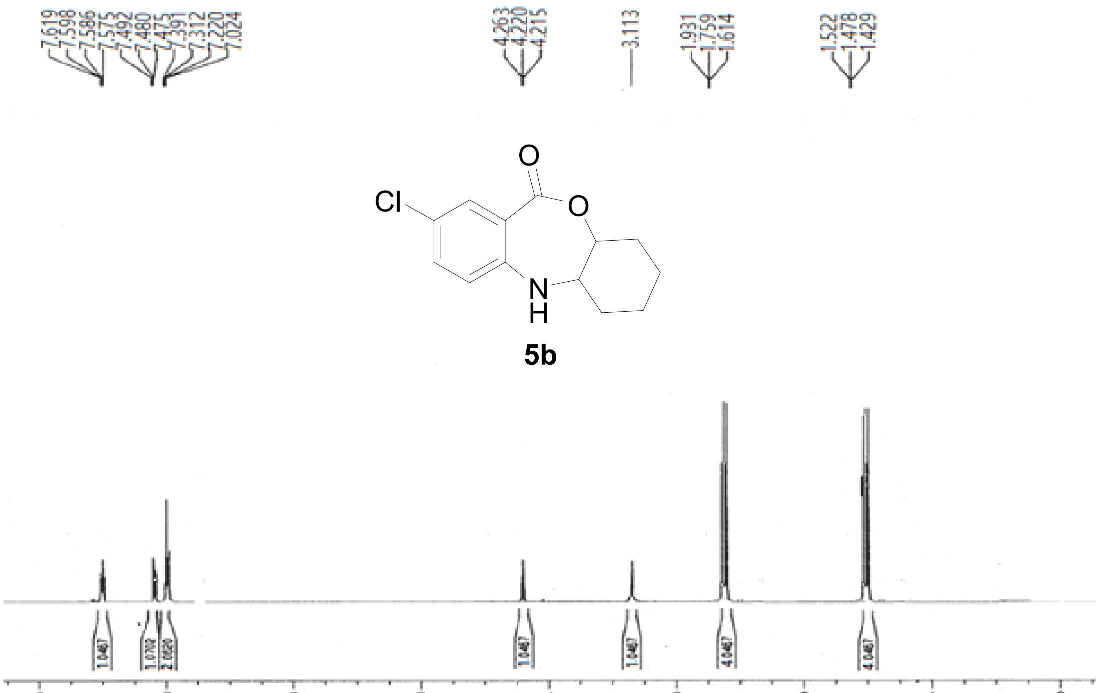

Figure S27. ^{1}H-NMR **5b**.

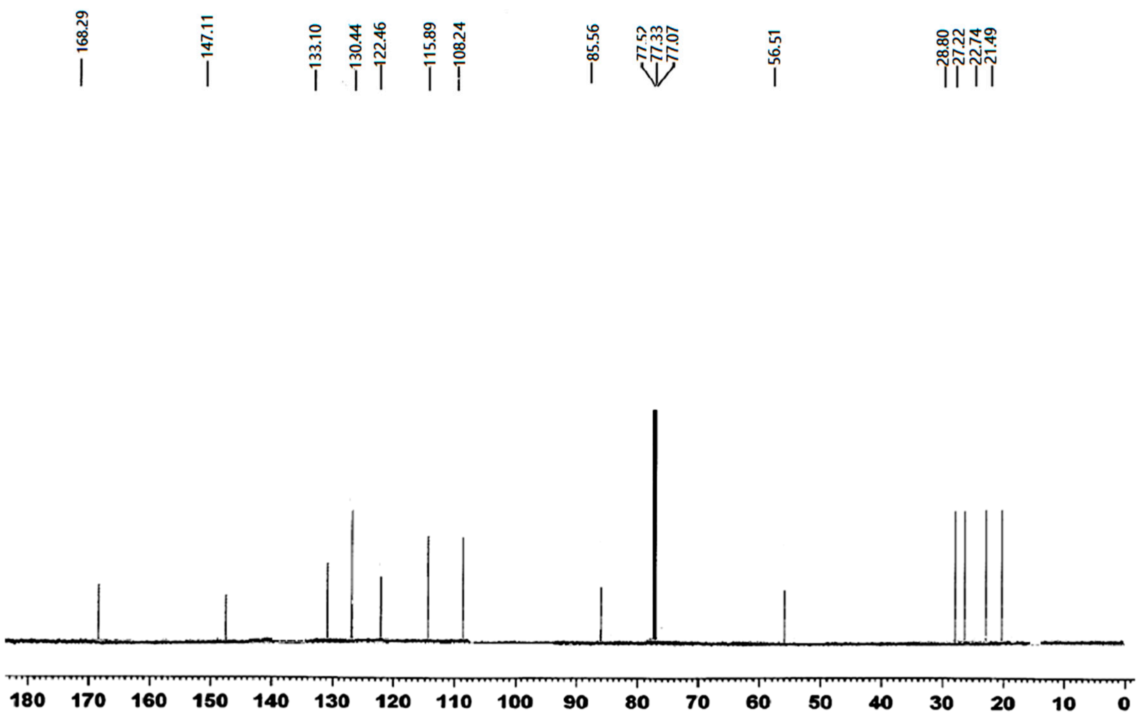

Figure S28. ^{13}C-NMR **5b**.

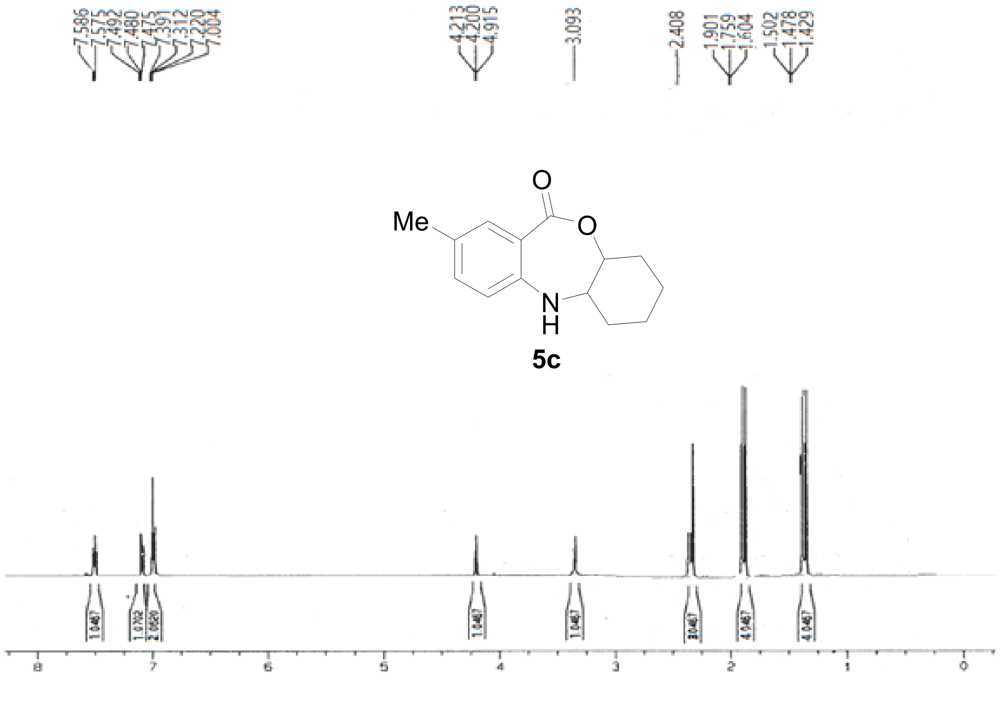

Figure S29. ^{1}H-NMR **5c**.

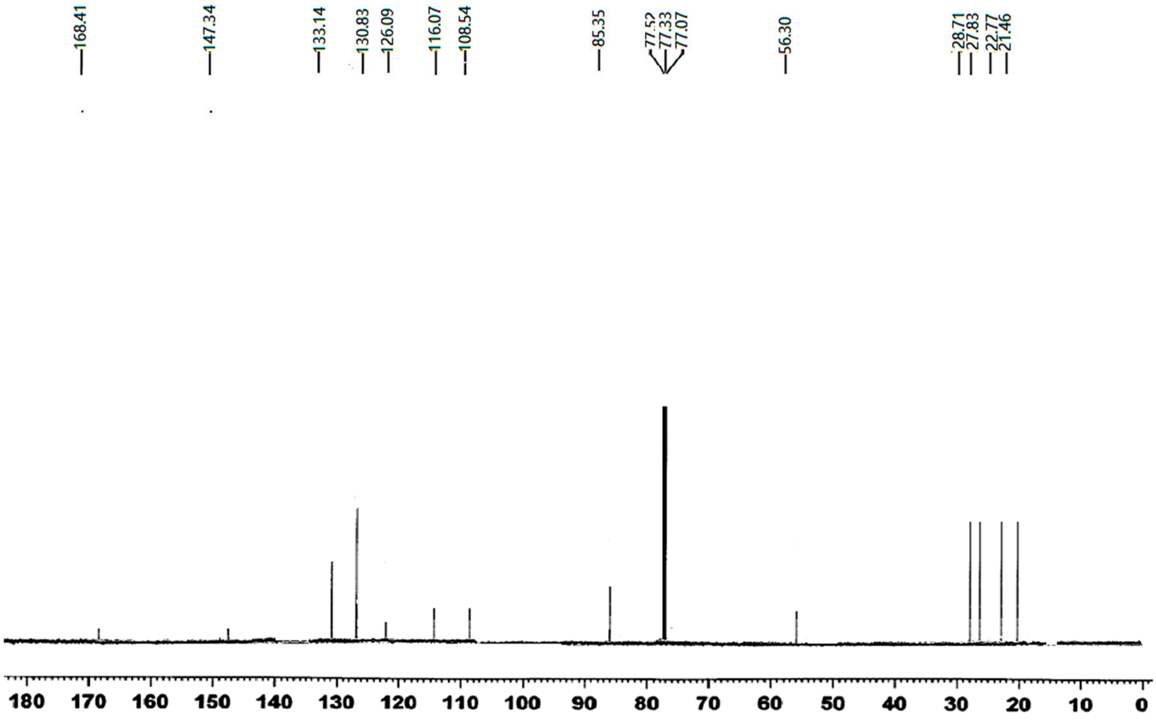

Figure S30. ^{13}C-NMR **5c**.
